# Mechano-regulation by clathrin pit-formation and passive cholesterol-dependent tubules during de-adhesion

**DOI:** 10.1007/s00018-023-05072-4

**Published:** 2024-01-13

**Authors:** Tithi Mandal, Arikta Biswas, Tanmoy Ghosh, Sreekanth Manikandan, Avijit Kundu, Ayan Banerjee, Dhrubaditya Mitra, Bidisha Sinha

**Affiliations:** 1 Department of Biological Sciences, Indian Institute of Science Education and Research Kolkata, Nadia, Mohanpur, 741246 India; 2 NORDITA, KTH Royal Institute of Technology and Stockholm University, Roslagstullsbacken 23, 10691 Stockholm, Sweden; 3 Department of Physical Sciences, Indian Institute of Science Education and Research Kolkata, Nadia, Mohanpur, 741246 India; 4 Present Address: Mechanobiology Institute, National University of Singapore, 5A Engineering Drive 1, Singapore, 117411 Singapore; 5 Present Address: Experimental Physics I, Universität Bayreuth, Universitätsstraße 30, 95447 Bayreuth, Germany

**Keywords:** Membrane homeostasis, Tension propagation, Excess area regulation

## Abstract

Adherent cells ensure membrane homeostasis during de-adhesion by various mechanisms, including endocytosis. Although mechano-chemical feedbacks involved in this process have been studied, the step-by-step build-up and resolution of the mechanical changes by endocytosis are poorly understood. To investigate this, we studied the de-adhesion of HeLa cells using a combination of interference reflection microscopy, optical trapping and fluorescence experiments. We found that de-adhesion enhanced membrane height fluctuations of the basal membrane in the presence of an intact cortex. A reduction in the tether force was also noted at the apical side. However, membrane fluctuations reveal phases of an initial drop in effective tension followed by saturation. The area fractions of early (Rab5-labelled) and recycling (Rab4-labelled) endosomes, as well as transferrin-labelled pits close to the basal plasma membrane, also transiently increased. On blocking dynamin-dependent scission of endocytic pits, the regulation of fluctuations was not blocked, but knocking down AP2-dependent pit formation stopped the tension recovery. Interestingly, the regulation could not be suppressed by ATP or cholesterol depletion individually but was arrested by depleting both. The data strongly supports Clathrin and AP2-dependent pit-formation to be central to the reduction in fluctuations confirmed by super-resolution microscopy. Furthermore, we propose that cholesterol-dependent pits spontaneously regulate tension under ATP-depleted conditions.

**Supplementary Information:**

The online version contains supplementary material available at 10.1007/s00018-023-05072-4.

## Introduction

Sensing and regulating the tension of the plasma membrane is crucial for cells to function effectively, especially when challenged by changes in the local micromechanical environment. Whether during malignant transformation, cytokinesis or morphogenesis, altered cell–cell and cell–substrate adhesivity are coupled to the dynamic cell signalling state defining these processes [28, 48]. A remodelled micro-environment can influence the cell membrane's mechanical state, changing its flexibility or tension. Processes involved in membrane homeostasis would ensure the maintenance of membrane tension or conservation of the microscopic fluctuations, providing the plasma membrane’s effective tautness or excess area (difference between microscopic area and projected area per unit microscopic area) [46, 64]. While enhancing tension can affect endocytosis [18, 29] and pit-formation [9], as displayed for clathrin-mediated endocytosis (CME) [2], conversely, cells can also alter their endocytosis/exocytosis rates to regulate tension. Studies have addressed the role of membrane trafficking in tension regulation, either in general [65] or focussing on particular pathways such as the CLIC-GEEC (CG) pathway [74], CME [17, 39] or those using caveolae [45]. Flattening of caveolae can regulate tension surge [69] suggesting that pits that participate in endocytosis may directly regulate tension.

Since pit-formation and internalization are common to all pathways and sometimes also regulated similarly, it becomes essential to understand whether the formation of endocytic pits in general can initiate tension regulation or their subsequent internalization is also essential. Addressing such questions would require mechanical perturbation of cells and following membrane mechanics and endocytosis state over time. Additionally, perturbing molecules necessary for pit formation or internalization can further elucidate their individual roles.

To quantify endocytosis, specific cargoes (e.g. Transferrin for CME) are usually labelled to quantify its corresponding pathways. However, Rab5 labels early endosomes of most pathways and, thus, reports the general state of endocytic trafficking [8]. A fraction of endosomes are recycled back to the plasma membrane using a fast-recycling arm, Rab4 [71] which can accumulate if either excess endosomes are formed, or the fusion with the plasma membrane is hampered [30]. Therefore, following labelled cargo (like Transferrin), as well as Rab5 and Rab4, together with well-established Transferrin uptake assays, can collectively describe the trafficking state of the basal plasma membrane.

For perturbing endocytosis, molecules utilized by multiple pathways can be targeted. For example, many constitutive pathways in HeLa cells (e.g. CME, caveolae) require dynamin for the scission of their endocytic pits, making it an important target. Dynamin's GTPase action can be inhibited by Dynasore, an agent that can prevent scission and, thus, internalization of pits in these pathways without affecting pit formation [40]. However, certain constitutive pathways, like CG, could still be operational even on Dynasore treatment—although its presence in HeLa cells is debated [75], while other dynamin-independent constitutive pathways are not well classified. Knocking down adaptor protein AP2 would directly impact the pit-formation step of CME [38, 50, 77]. Cholesterol is required very early for the primary clustering of lipids/proteins in several pathways [32]. The formation of endocytic carriers critically requires cholesterol for pathways such as CG pathway and caveolae, while assembly of clathrin and membrane curving in CME mostly requires adapters like AP2 [11, 27, 77]. Depletion of Cholesterol (by MβCD) thus, can arrest the formation of new pits of the CG pathway and caveolae. However, it can also enhance tension [7], and has been reported to cause a substantial reduction in the formation and budding of deep pits of CME without completely blocking CME [2, 4, 58, 73]. The use of ATP-depletion can reduce both the pinching-off of a majority of pathways as well as the formation of pits in pathways like CME [62] and caveolae [69]. Thus, ATP depletion could suppress the completion of a multitude of constitutive endocytic pathways and, therefore, could be used to study the role of scission in general. The alterations of these endocytic events need to be understood in response to perturbations to the mechanical microenvironment.

Mounting evidence [19, 36, 79] shows significant crosstalk between endocytic machinery and cell adhesion molecules and its role in regulating cellular homeostasis [80]. While cell–substrate adhesion profiles of single cells have been reported to determine the distribution of endocytosis sites [22], studies have also addressed if de-adhesion can trigger mechano-regulation via endocytosis [74] or blebbing [49]. Although measurements of apparent tension have been reported during cell spreading [20, 69] similar studies involving tension measurements during de-adhesion have not been performed, to the best of our knowledge. Measurement of membrane mechanics can be either done at specific points by optical tweezer-based tether pulling or by studying the spontaneous fluctuations of the membrane measured using interference reflection microscopy (IRM). Several studies [7, 66] have proposed spatial heterogeneities in tension. Multi-point measurements would enable accessing the local distribution of excess membrane undulations or tension in cells during different phases of de-adhesion.

In this study, we address the contribution of endocytosis to mechano-regulation during de-adhesion by employing the IRM-based measurement of effective fluctuation–tension of de-adhering HeLa cells. IRM [1, 10, 12, 21, 37, 54] primarily provides information about the distance of the basal membrane from the substrate—also called the membrane ‘height’. Spatio-temporal measurements of height provide fluctuation amplitude (SD_time_) excess area and enable deriving mechanical parameters like fluctuation-tension [7, 67]. Spatial maps of fluctuation-tension (also termed tension in the rest of the manuscript) and their temporal evolution help us quantify the changes.

We use total internal reflection fluorescence (TIRF) microscopy to image Rab5 and Rab4 labelled structures, labelling the early endosomes and rapidly recycling endosomes, respectively. This measures how de-adhesion alters internalization during endocytosis. Fluorescent cargo—transferrin—is used to further validate the involvement of endocytosis while Dynasore, dynamin-mutants, knockdown of AP2, Cholesterol and ATP depletion are used to perturb steps/pathways of endocytosis to assess their contribution to the mechano-regulation.

## Results

### De-adhesion-mediated increase in membrane fluctuations is actin-dependent

IRM images reflect the distance of the cellular basal membrane from the glass coverslip and, thus, can be used to study the spatiotemporal basal PM height fluctuations in adherent cells [6]. Qualitatively, darker regions were closer to the coverslip while intensity increases with height till ~ 100 nm and periodically oscillates. Calibration with standards (beads) was performed to quantify the relative heights, followed by selecting regions where the intensity-height conversion was possible—usually, regions that are ~ 100 nm from the coverslip and fall under the first branch of the intensity profile (that displays bands) [6]. The selected regions (square membrane patches) were termed first-branch regions (FBRs). Multiple such regions were marked for every cell.

The time series of membrane height fluctuations (obtained from single pixels) were used to get mean height and standard deviation (SD) of height and termed SD_time_. Pixel-wise measurements were averaged over neighbouring pixels to get “FBR-wise” data. SD_space_ was obtained from a snapshot—comparing height across NxN pixels of any FBR where N was usually 12 in this study (see "Methods"). These parameters depicted the amplitude of fluctuations. Averaging over all FBRs in a cell and pooling such data from all cells provided the “cell-wise” data. We compared these parameters between control and treated cells to understand how a mechanical perturbation like de-adhesion altered membrane mechanics.

IRM provides the ability to map fluctuation-tension, get its distribution in the cell, and thus differentiate between large global and local changes. In this study, we have compared changes at the level of whole cells (termed cell-wise) and those that show up in the local distribution of tension in single cells. However, while comparing local data, to ensure that the repeated measurements per cell do not falsely strengthen the statistics, we have employed linear mixed models (LMM) as usually used [24, 56] to account for the nested grouping of replicate measurements.

Finally, it is important to note certain limitations. Fluctuations reported by IRM are mainly thermal but can have contributions from active (ATP-dependent, non-thermal) processes in cells [76]. Using a model-free method, a recently developed algorithm [43, 44], quantified the effect of active forces on membrane fluctuations (termed activity) and revealed local membrane fluctuations to be weakly active. However, fluctuation-tension solely should not be used for drawing inferences.

We deal with this primarily by using direct measurements of fluctuations amplitude. We also compare activity at different time points of de-adhesion to record the level of deviation of the measured fluctuations from equilibrium. Finally, we corroborate the main effect of de-adhesion on fluctuation-tension with apparent membrane tension measurement using the generally accepted optical-trap-based tether extraction method.

**Fig. 1:**
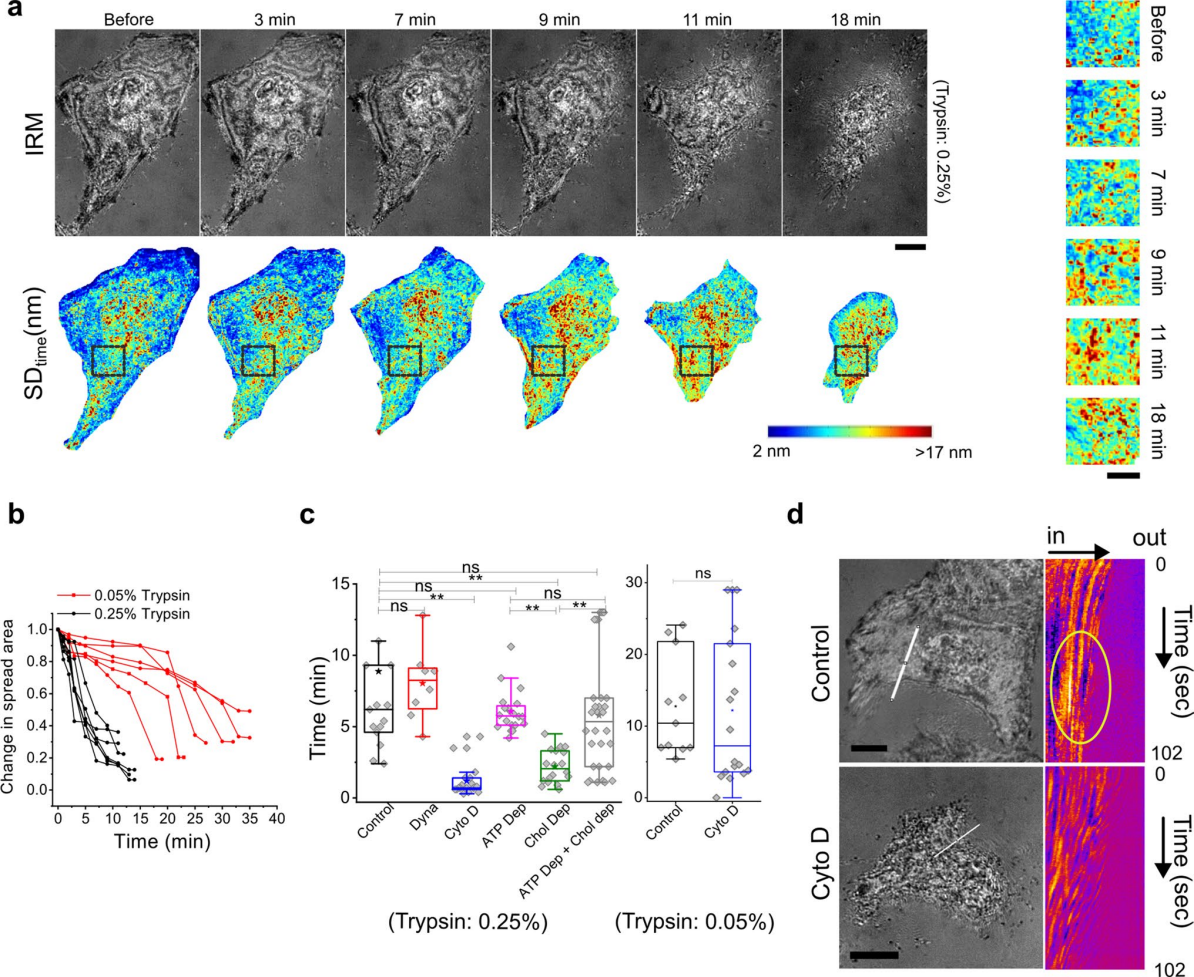
Phases and variability in de-adhesion. **a** IRM images of a representative cell before and after Trypsin–EDTA addition at 0.25% (faster) concentrations and corresponding temporal fluctuation maps. The time after de-adhering solution addition is mentioned. Scale: 10 μm. Zoomed-in views of SD_time_ maps (right). **b** Profiles of spread area for 6 representative cells in each concentration with time after Trypsin–EDTA addition in the two different concentrations. **c** Time taken to de-adhere 67% of spread area of cell. N_cell_: Control = 15, Dyna = 8, Cyto D = 17, ATP Dep = 20, Chol Dep = 18, ATP Dep + Chol Dep = 11. Control = 11, Cyto D = 18. **d** Representative colour-coded kymographs of IRM intensity of Control and Cyto D-treated cell. ROIS drawn perpendicular to the de-adhering front of a cell. Scale: 10 μm

HeLa cells were treated with either a low (0.05% Trypsin–EDTA) or high concentration (0.25% Trypsin–EDTA) of de-adhering solution and imaged by IRM (Fig. 1a) to study the changes in membrane fluctuations and mechanics in the basal membrane during de-adhesion. We observed high variability in the time cells took to de-adhere (Fig. 1b). To quantify the rate of de-adhesion, the time taken by each cell to reduce their spread area to 67% of the initial was calculated (Fig. 1c). We found that, among the various treatments used, Cytochalasin D (Cyto D)—an agent reducing actin polymerization [61] of the actin cortex resulted in a very fast de-adhesion. Therefore, a lower concentration of de-adhering solution of Trypsin (0.05%) was used for Cyto D experiments. This was in line with the understanding that during de-adhesion, the contractile cortex caused lateral retraction [34, 63]. At lower trypsin concentrations, the process could be slowed down such that the decay times for Control and Cyto D were similar (Fig. 1c).

**Fig. 2:**
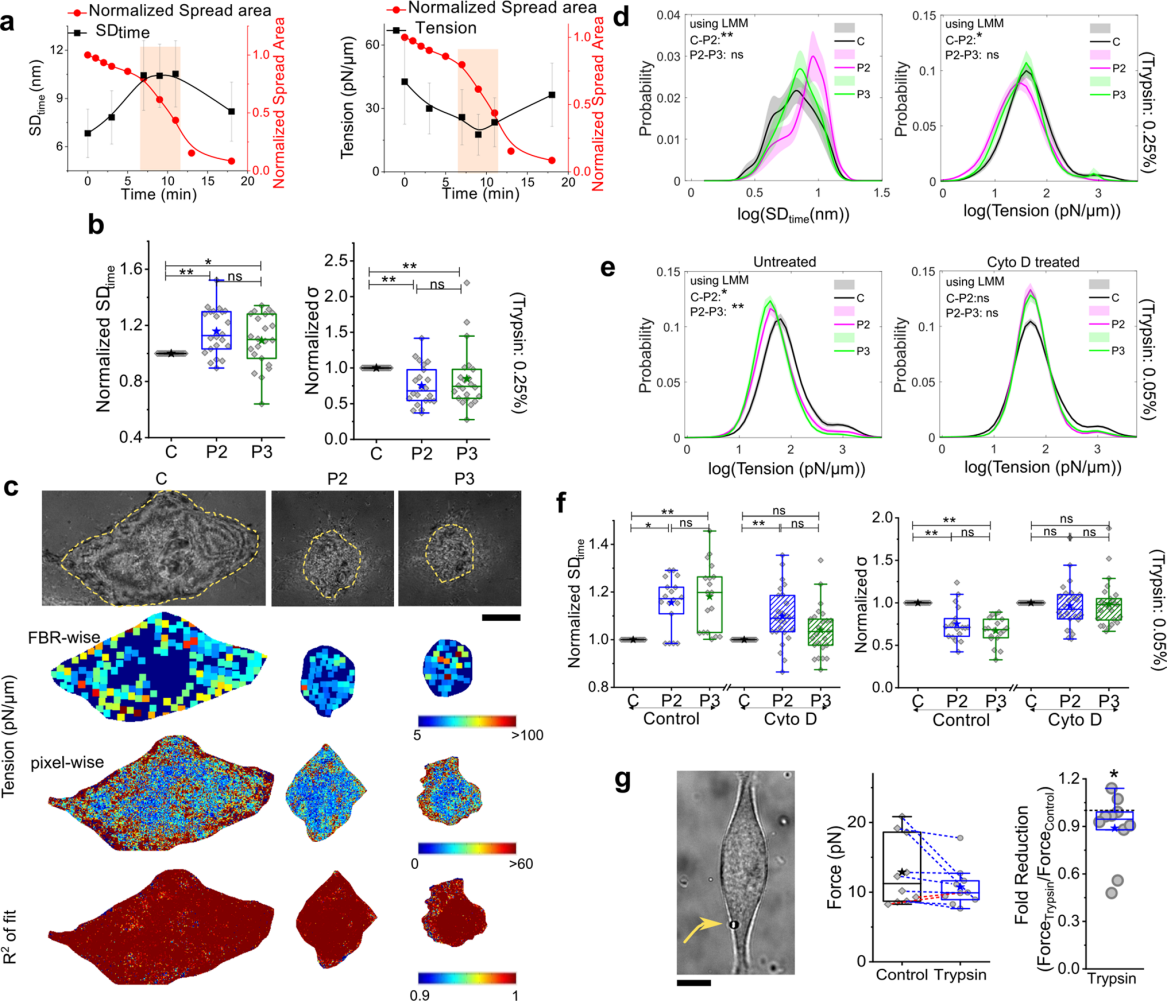
Membrane slacks transiently.** a** Representative time series of indicated parameters for a single cell during fast de-adhesion. **b** Comparison of normalized amplitudes of fluctuations and tension following the same cells across the indicated phases of de-adhesion. Data represent average median values calculated for 22 cells using FBRs of sizes 0.75 μm^2^. Normalization is performed by dividing any particular cell’s measurement at P2 or P3 by the measurement at C. Table S1 provides the minimum number of FBRs used per cell in each mechanism used for calculating the average. **c** IRM images of a representative cell at different phases of de-adhesion, and the corresponding FBR-wise tension values mapped back on the cell outline. The dark blue background represents the cell, and coloured boxes denote tension values derived from averaged PSDs from the indicated regions. Lower panel indicates pixel wise tension map and corresponding *R*^2^ map. **d** Comparison of probability of logarithm of temporal fluctuation and tension across the de-adhesion phases using 0.25% Trypsin–EDTA. Distributions were obtained from FBR-wise values for each cell and averaged. Shaded regions denote the SEM. **e** Comparison of probability of logarithm of tension measures across the cells (FBR wise) across the de-adhesion phases of Control (left) N_cell_ = 18, and Cytochalasin D (right) treated cells N_cell_ = 25 using 0.05% Trypsin–EDTA. **f** Comparison of fold change in the mean amplitude of fluctuations and median tension for the same cells followed over time of Control and Cyto D-treated cells. Normalization is performed by dividing any cell's measurement at P2 or P3 by the measurement at C. **g** Left: Typical image of a cell used for the tether-pulling experiment. N_cell_ = 10. The trapped bead is marked out with an arrow. Right: Fold reduction of force after Trypsin addition (higher concentration: 0.25%). Black *denotes Mann–Whitney *U* statistical significance test with Bonferroni correction is performed, * denotes *p* values < 0.016, and **denotes *p* value < 0.001. Scale bar = 10 μm

**Fig. 3:**
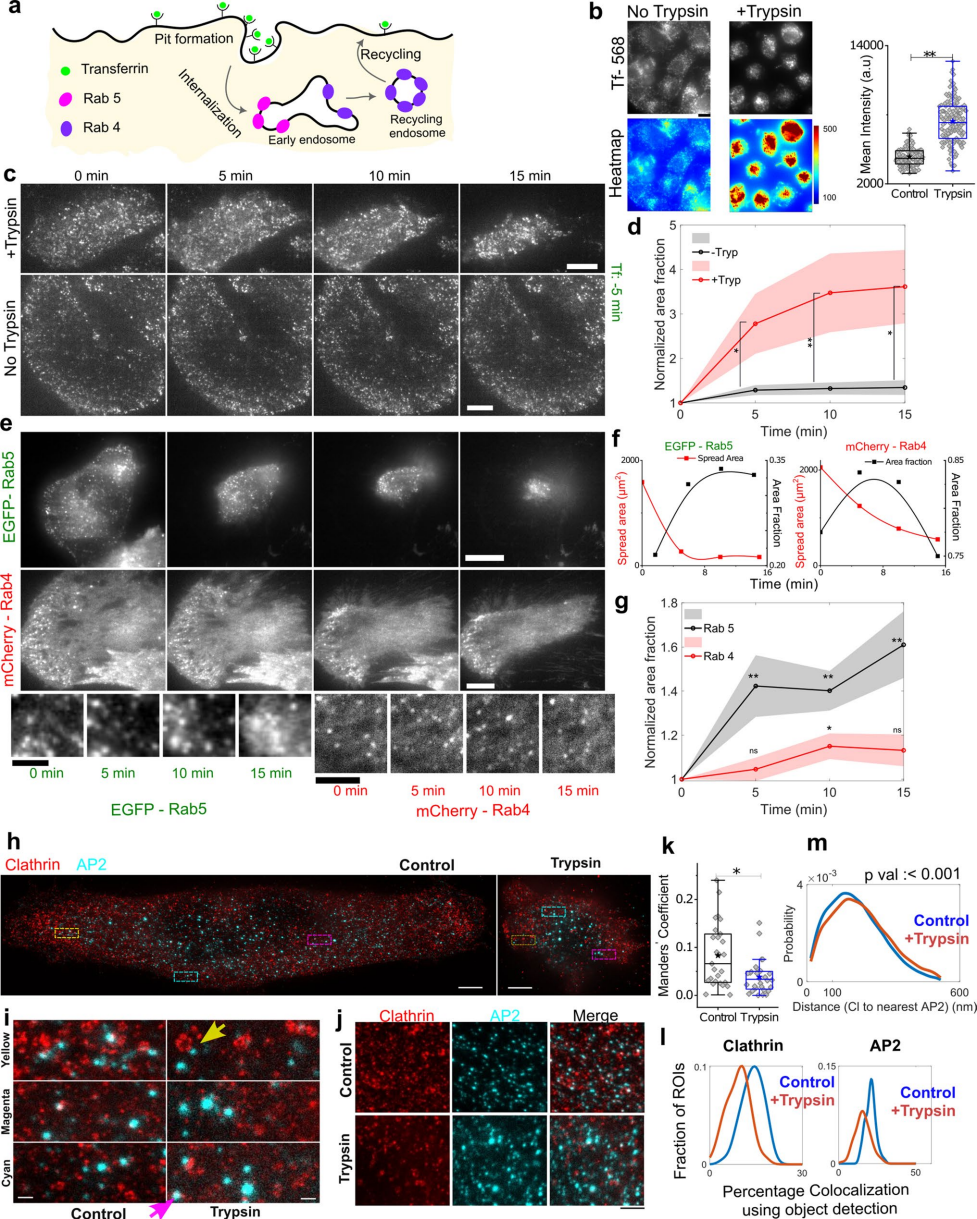
De-adhesion induces endocytosis. **a** Schematic of formation of early endosomes and maturation into recycling endosomes. **b** Transferrin uptake assay in Normal and 0.25% Trypsinised cells N_cell:_ Control = 116, Trypsinised = 125. **c** Representative TIRF images of same HeLa cells followed through time, puncta marked with Transferrin- 568 with 0.25% Trypsin (upper panel), and without Trypsin (bottom panel). Scale bar = 10 µm. **d** Normalized area fraction of Transferrin-marked puncta followed through time with and without Trypsin. N_cell:_ Trypsin = 53, without Trypsin = 16. Shaded regions denote SEM measure while average per distribution. **e** Representative TIRF images of the same HeLa cells transiently expressing EGFP -Rab5 (upper panel) or mCherry -Rab4 (lower panel) before (0 min) and after administration of de-adhesion media. Bottom panel represents zoomed-in view (Scale bar = 5 µm). **f** Change in area fraction of Rab5 (left) and Rab4 (right) as spread area reduces on de-adhesion for typical single cells. **g** Normalized area fraction of Rab5 and Rab4 through different time points before and after addition of 0.25% Trypsin. N_cell:_ Rab5 = 15, Rab4 = 43. Shaded regions denote SEM. Table S1 provides the list of the number of cells and other statistical parameters. **h** Representative STED images of Clathrin (Clathrin heavy chain) and AP2 (α subunit) Scale bar = 3 µm. **i** Zoomed in sections (ROIs marked in h with colours as indicated) Scale bar = 100 nm. Yellow arrow-head points at edge-localized AP2 next to well-matured calthrin-coated pit with distinctly distributed clathrin. Pink arrow-head points out smaller clathrin puncta much better colocalized. **j** Other randomly selected ROIs from other cells. Scale bar = 1 µm **k** Quantification of colocalization using Mander’s coefficient from 10 cells and 27 ROIs for Control, and 20 cells and 32 rois for + Trypsin condition. **l** Colocalization from object detection from N_cell_ = 15 and 28 for Control and trypsinized conditions with 70 and 75 ROIs, respectively. **m** Comparison of distance between clathrin punctas and the nearest AP2 puncta for only those pairs that lie within 525 nm of each other. > 40,000 clathrin puncta and > 14,000 AP2 puncta were used. Data (h-m) are representative of 3 independent sets with 45 and > 60 cells imaged in each condition

**Fig. 4:**
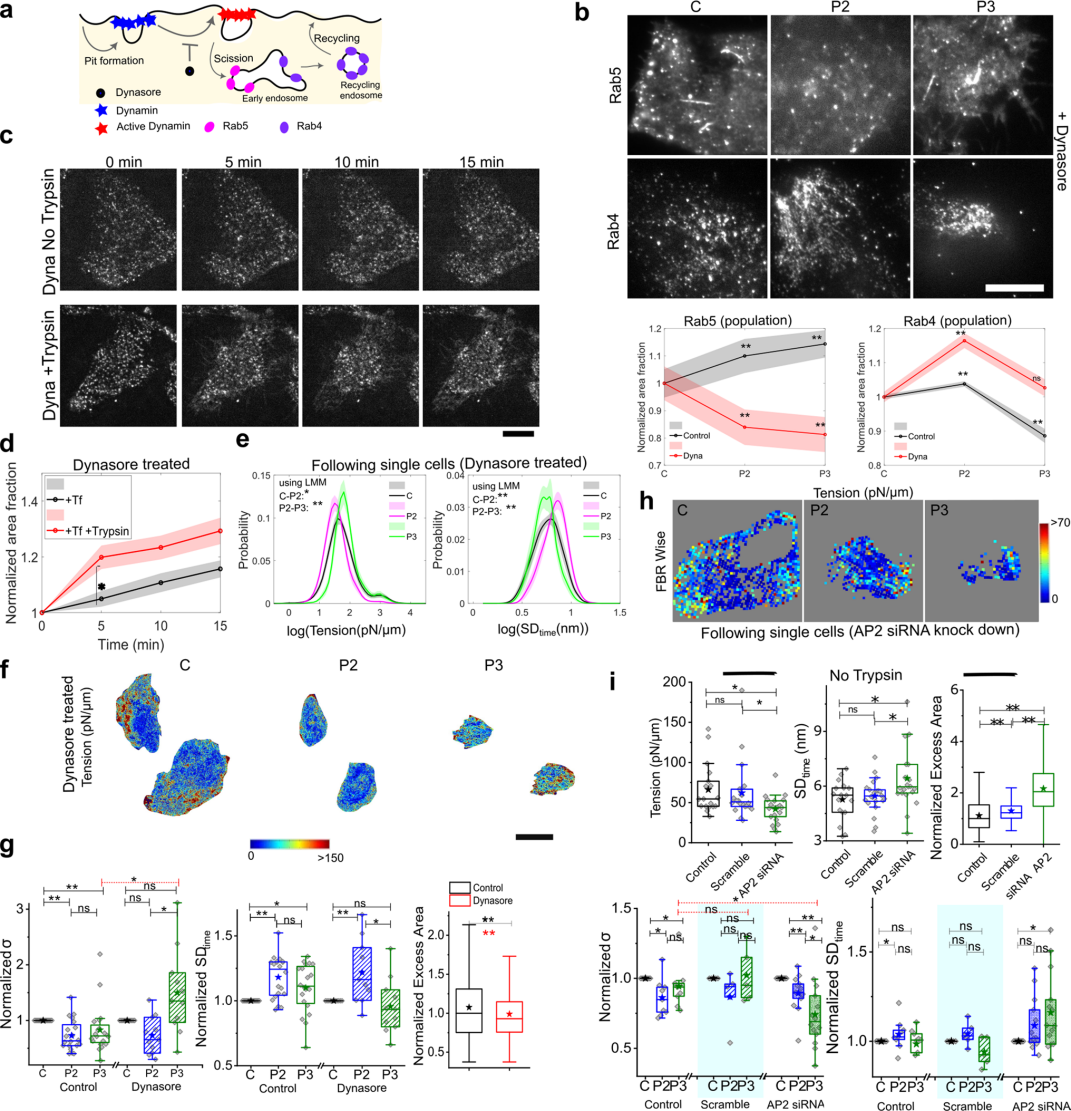
Blocking endocytosis does not stop tension recovery. **a** Schematic diagram of pit formation, scission, recycling and inhibition of dynamin. **b** TIRF images of Dynasore-treated cells transfected with EGFP-Rab5 or immune-stained with Rab4 before and after de-adhesion. Scale bar = 10 μm. Bottom: Normalized area fraction of Rab5 marked early endosomes (left) and Rab4 marked recycling endosomes (right) of Control and treated with Dynamin inhibitor- Dynasore. N_cells(Rab5):_ Control ~ 57, Dynasore ~ 43, N_cells(Rab4):_ Control ~ 70, Dynasore ~ 103. **c** Representative TIRF images of Dynamin-inhibited HeLa cells followed through time, puncta marked with Transferrin- 568 without Trypsin or with 0.25% Trypsin. Scale bar = 10 µm. **d** Normalized area fraction of Transferrin-marked puncta followed through time with and without Trypsin. N_cell_: Trypsin = 12, without Trypsin = 8. Shaded regions denote SEM. **e** Probability distribution of FBR wise log temporal fluctuations and tension of Dynasore treated same HeLa cell followed through different phases of De-adhesion. N_cell:_ Dynasore = 11. Shaded regions denote SEM. **f** Typical tension map of Dynasore-treated cells in the different phases of de-adhesion. Scale bar = 10 μm. (Upper). **g** Fold change in cell-averaged parameters comparing each cell with its own measurements at different phases. N_cell:_ Control = 20, Dynasore = 11. For excess area, FBR-wise comparison is presented with red *denoting statistical significance obtained from LMM. **h** FBR-wise tension map of a representative AP2 knockdown cell in different phases. **i** Top: Cell-wise comparison of tension and SD_time_ of Control, scramble (scrambled siRNA), AP2 siRNA-treated cells without de-adhesion. For excess area, FBR-wise comparison is presented. Bottom: Fold change in cell-averaged parameters comparing each cell with its own measurements at different phases of de-adhesion. N_cell:_ Control = 9, Scramble = 5, AP2 siRNA = 14. *n* = 3 independent experiments. One-way ANOVA with Bonferroni correction is performed for SD_time_ since the data are normal. For tension, the Mann–Whitney *U* test is performed and *denotes a *p* value < 0.016 (adjusted by group size of 3 per experiment). *N* = 3 independent experiments. Table S1 provides a list of the number of FBRs

**Fig. 5:**
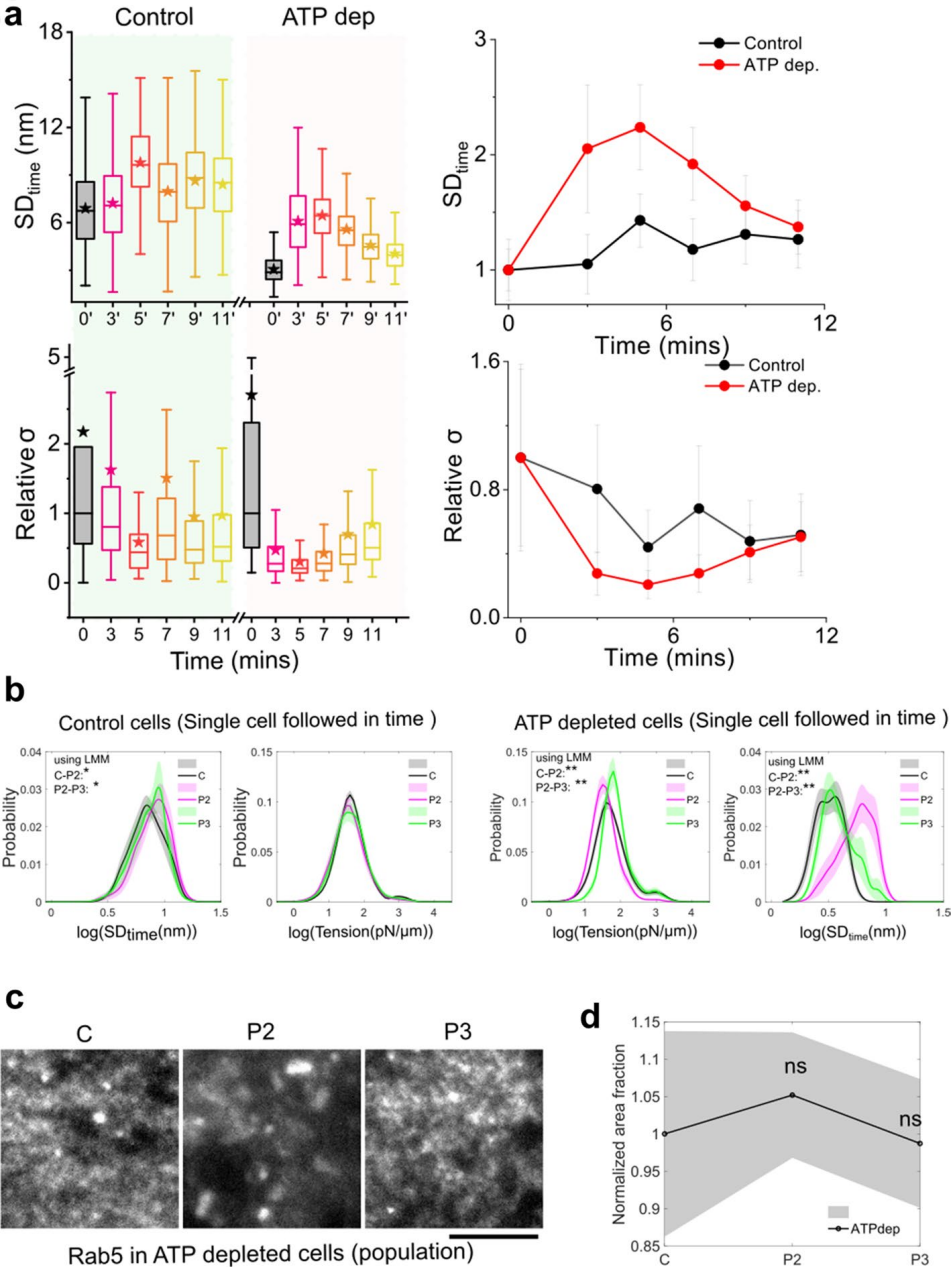
Tension recovery can start without ATP. **a** Time series boxplots and median (with median absolute deviation (MAD) as error bar, lower panel) for different parameters for Control and ATP-depleted cells on de-adhesion using an FBR size of 4.67 μm^2^. N_cells(control)_:6 N_cells(ATP Depleted)_: 9. **b** Probability distribution of FBR-wise log temporal fluctuations and tension of Control and ATP depleted same HeLa cell followed through different phases of De-adhesion. Shaded regions denote SEM. **c** Zoomed-in TIRF images of different cells (transfected with EGFP-Rab5) at different stages of de-adhesion in ATP-depleted condition. **d** Normalized area fraction of Rab5 in ATP-depleted condition. Shaded regions denote SEM. N_cells_: C = 26, P2 = 41, P3 = 39

**Fig. 6:**
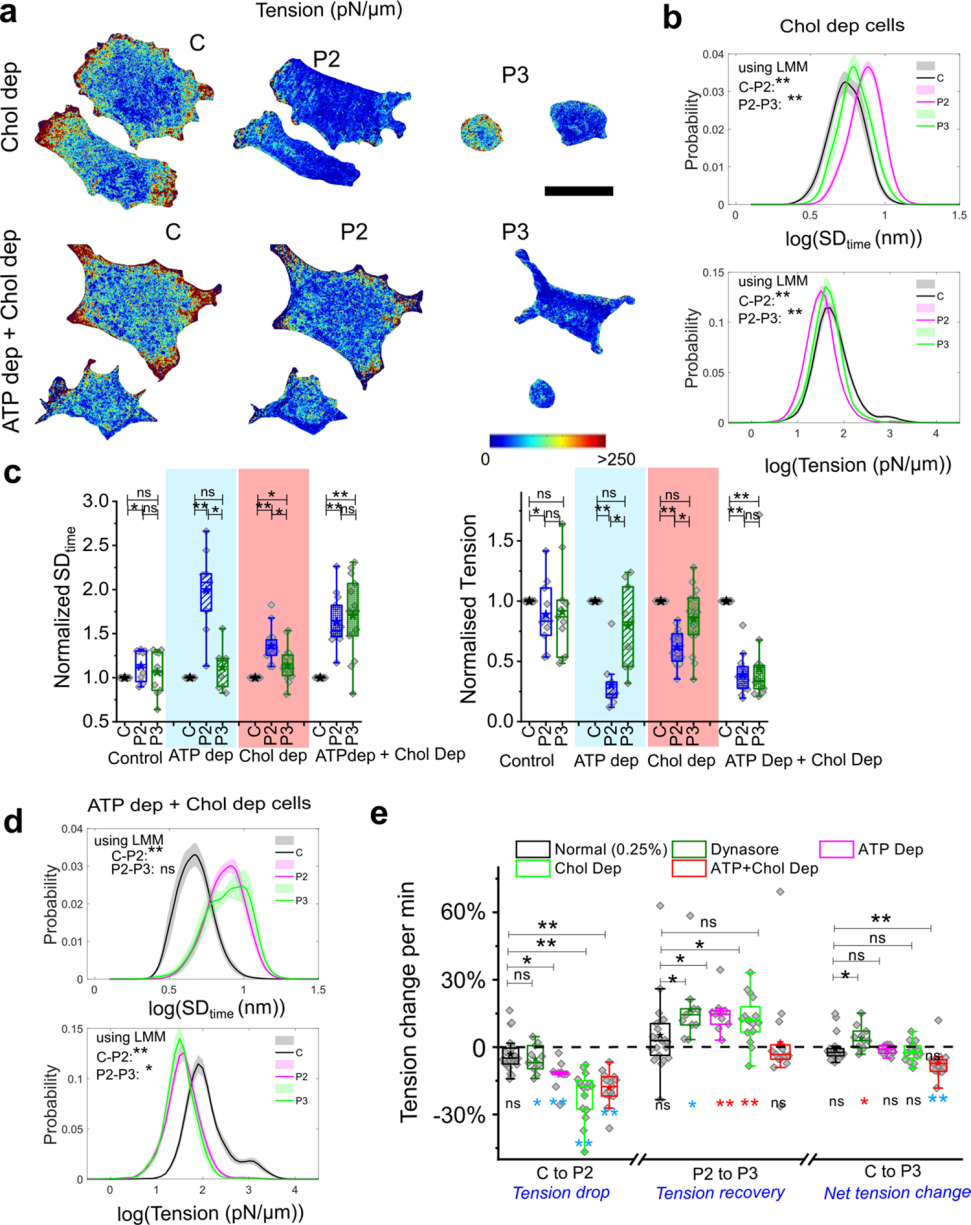
Passive regulation is cholesterol-dependent. **a** Representative tension maps of cholesterol-depleted (Upper Panel) and ATP-depleted as well as cholesterol-depleted cells (Lower Panel) in three phases of de-adhesion**.** Scale bar = 10 μm. **b** Probability distribution of FBR-wise values of log temporal fluctuations and tension of cholesterol-depleted HeLa cell followed through different phases of de-adhesion. Shaded regions denote SEM. **c** Fold change in cell-averaged parameters comparing each cell with its own measurements at different phases. N_cells_: Control = 11, ATP dep = 9, Chol dep = 16, ATP dep + Chol dep = 15. **d** Probability distribution of FBR-wise values of log temporal fluctuations and tension of ATP depleted as well cholesterol-depleted same HeLa cells followed through different phases of de-adhesion. Shaded regions denote SEM. **e** Plot of rate of fractional change of tension when cells transit between different phases (mentioned). One-way Anova with Bonferroni correction is performed for SD_time_ since the data are normal. For tension, the Mann–Whitney *U* test is performed and *denotes *p* value < 0.016 (adjusted by group size of 3 per experiment). *n* = 3 independent experiments. N_cells_ mentioned in Table S1

**Fig. 7:**
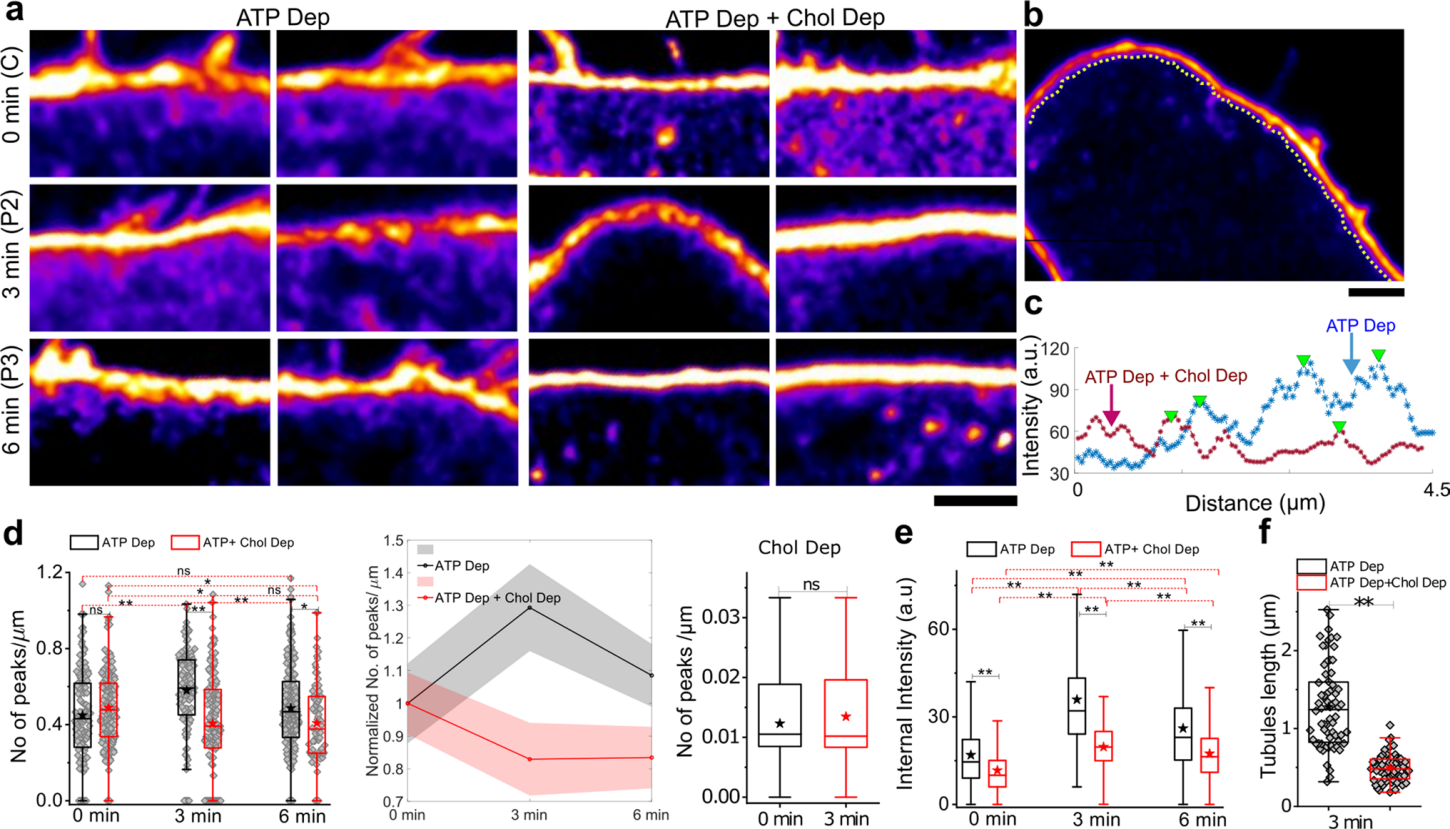
Membrane imaging reveals cholesterol-dependent tubules. **a** Representative zoomed in colour-coded confocal images of cells [ATP-depleted (left) and ATP-depleted as well as cholesterol-depleted (right)]. Scale bar = 2 μm. **b** Typical line scans are performed parallel to the membrane on the cytosolic side. Scale bar = 2 μm **c** Intensity profile of typical line scans of ATP-depleted and ATP Dep + Chol Dep condition with triangles pointing out detected peaks with minimal width and height. **d** Box plots (left) and line plot (centre) comparing number of peaks/μm in ATP-depleted and ATP + Cholesterol depleted through different time points of de-adhesion. Number of cells = 20, 18, 28 for 0, 3, 6 min, respectively (ATP-depleted), 21, 24, 21 for 0, 3, 6 min, respectively (ATP + cholesterol-depleted). Effect of de-adhesion on only cholesterol-depleted cells were evaluated using 97 and 113 ROIs from 8 and 9 cells. **e** Comparison of intensity detected per μm of various 4 μm lines drawn as explained in (**b**). **f** Evaluation of length of tubules using analysis of 68 and 58 ROIs from 18 and 15 cells of ATP-depleted and ATP + Cholesterol-depleted cells, respectively. **denote *p* value < 0.001 calculated using Mann–Whitney *U* test. Table S1 lists the number of peaks

In general, de-adhesion caused an increase in temporal fluctuations (Fig. 1a—7 min), followed by lateral retraction (Fig. 1a—9–18 min). The increase in fluctuation amplitude (SD_time_) could be visualized from the maps (Fig. 1a bottom, right). Following the lateral retraction of the edge using a kymograph) of the IRM intensity (along the white line, Fig. 1d), we found that as the edges retract inwards, intensity patterns lying inward also moved. Membrane height (IRM intensity) increased in a cluster close to the edge (white oval, Fig. 1d) as the retraction progressed. This accumulation faded away with time (section below the oval, Fig. 1d). While the membrane undulations were locally enhanced in control cells, such local increase was less prominent in Cyto D-treated cells (Fig. 1d). Thus, as de-adhesion progressed, cytoskeleton-dependent transient accumulation of membrane folds point to the ongoing membrane remodelling.

We proceeded to measure the fluctuations and effective membrane-mechanical parameters from the fluctuations during the different phases and over time to underpin the effect of de-adhesion on membrane mechanics quantitatively.

### The initial rise in fluctuations is regulated back

Since the rates of de-adhesion in cells were different, we classified the data in this and the following sections based on the level of reduction in spread area. For every cell, we divided the process of de-adhesion into four phases. The “C” phase was defined as the period for which the cell had not been treated with the de-adhering agent (Trypsin). The “P1” phase was demarcated as the slow de-adhesion phase before the exponential fall of the spread area started. Typically, the spread area in this phase remained within 90% of the initial cell spread area. The “P2” phase was defined as the period when the spread area exponentially reduced, at least to ~ 67% of the original value. The “P3” phase marked the period when the low spread area had stabilized to ~ 40–20% of the initial (Fig. 1b).

Height fluctuations were measured before (phase: C) and then every few minutes after adding de-adhering medium from image stacks acquired at every time-point, where each movie lasted for ~ 102 s. Only membrane regions that remained adhered through the movies were analyzed. On following a representative cell in time (Fig. 2a; same cell as shown in Fig. 1a) or averaging over a population (Fig. 2b), we found that the amplitude of temporal height fluctuations (SD_time_) first increased, followed by a decrease/saturation on de-adhesion. The reverse was observed for fluctuation-tension (Fig. 2a). Maps of FBR-wise fluctuation-tension (Fig. 2c) revealed the lowered tension state of the cell followed by an increase in the regions that remained adhered. Strictly for visualization purposes, we also created a pixel-wise map of tension (Fig. 2c). Mapping tension showed a reduction in the initial heterogeneity when cells are at phase P2. The global trend (Fig. 2b**)** was corroborated by the distribution of local (FBR-wise) fluctuation amplitude and tension for single cells. Clearly, the changes were greater than the error calculated while averaging the distributions over multiple cells and repeats (Fig. 2d).

We confirmed that in the absence of de-adhesion media, the fluctuations or tension of these cells did not change over 20 min (Fig. S1). Following the same regions in single cells through de-adhesion also showed the dip and recovery of tension (Fig. S2), confirming that regions that remain adhered also underwent these changes. Although the spatial height variation (SD_space_) and excess area showed a decreasing trend in contrast to SD_time_ (Fig. S3a), using a gentler substrate detachment reagent—TrypLE (Fig. S3b) or at lower trypsin concentration, their trends matched [Fig. S3 c, d (0.05% Tryp)].

The corresponding entropy-generation rate obtained from fluctuations of de-adhering cells changed mildly (Fig S3a), implying that the measured fluctuations did not capture any major enhancement in non-equilibrium activity in the frequencies assayed. Since actin remodelling was expected during de-adhesion, its impact on tension reduction was next studied.

### The initial rise in fluctuations is weaker on cortex disruption

We used Cyto D to weaken the cortical actin. At 0.05% Trypsin, the spread area reduction rate was similar for Control and Cyto D, but cells de-adhered properly. Tension reduction in P2 was not substantial in the presence of Cyto D or reduced filamentous cortical actin (Fig. 2e, f). Although fluctuations were enhanced weakly (than Control (Fig. 2f, S4) at P2, by P3, unlike in Control, Cyto D showed similar fluctuations as C. In line with Fig. 1d, these data also suggest that disruption of cortical actin reduced membrane accumulation or enhancement of fluctuations.

De-adhesion only occurs at the basal membrane. To study its effect on the apical membrane, we next extracted membrane tethers from cells and measured tether forces. These were measured on single cells—before and after initiating de-adhesion (Fig. 2f top, Fig. S5) within the first—3–7 min. There was a reduction in force for 8/10 cells, which showed a 2–52% reduction in force (or effectively a 4–77% reduction in apparent tension).

Next, we examined whether the tension reduction and subsequent regulation correlated with endocytic activity.

### De-adhesion increases early endocytic and fast recycling endosomes near the basal plasma membrane

To quantify endocytosis, we utilized three strategies—labelling the cargo of an endocytic pathway, labelling early endosomes using Rab5 and labelling recycling endosomes with Rab4 (Fig. 3a). For analyzing fluorescent puncta imaged in TIRF, the puncta were detected as objects and analyzed (Fig S6a). Since nearby objects could be distinguished from connected ones, we scored for the area fraction or the area covered by the puncta per µm^2^ of the analyzed area. At first, we followed a cargo of CME—Transferrin (Tf)—added in Control and de-adhering cells (Fig. 3b, c) and found a clear increase in Tf's internalization (Fig. 3b) as well incorporation (per μm^2^) in the plasma membrane as clusters in de-adhering cells (Fig. 3c, d, Fig S6 b–d). Analyzing their disappearance indicated enhanced short-time dynamics on de-adhesion (Fig. S6. E–i) validating increase in pits not plaques [60].

The involvement of the endocytic machinery was next confirmed by labelling early endosomes by Rab5 [8] and the short-loop (fast) recycling endosomes by Rab4 [71]. Near the plasma membrane, endosomes mainly contain these two labels [71]. The Rab4-containing endosomes emerge from the same endosomes as Rab5 [71] and thus are studied to evaluate the state of endocytic and recycling activity. We imaged EGFP-Rab5 and Rab4 -mCherry using TIRF microscopy (Fig. 3e, f, Fig S6). The area fraction of early endosomes (Fig. S6), calculated for whole cells, showed an increase and a subsequent tapering off/decrease (Fig. 3f). The increase was anti-correlated initially with the reduction in spread area (Fig. 3f). However, it was difficult to discount the effect of heterogenous de-adhesion in the observed trend since some regions had denser features than others. Thus, we looked at regions that stayed on through the observed time scale (15 min). We followed the area fraction of Rab5 for the same sub-cellular region of interest (ROI) (Fig. 3g). The rise and saturation were found to be consistent (Fig. 3g).

To further validate if endocytosis was ramped up, we analyzed clathrin-coated pits (Fig. S6j). Imaging cells fixed before or after faster de-adhesion. Although an increase and saturation in the area fraction of these pits were observed, the changes were not appreciable. However, on evaluating with super-resolution using Stimulated Emission Depletion (STED) microscopy, we found that clathrin was less colocalized with its adaptor AP2 (Fig. 3h) on de-adhesion. As reported earlier, AP2 was observed either well-colocalized with smaller clathrin clusters or at the edges of larger structures (Fig. 3h, zoomed-in sections) and expected to be more curved [70]. On increasing tension, previous studies report a higher fraction of AP2 with clathrin indicative of flat structures [9]. Our observation of AP2 localized at edges (Fig. 3i, j) and showing lesser colocalization (Fig. 3k, l) might indicate that more fraction of clathrin structures are pits on de-adhesion. We also find an increase in the distance between clathrin and AP2 clusters after de-adhesion, comparing pairs already with 525 nm of each other (Fig. 3m) or higher. The significance holds true even while comparing pairs within 165 nm but does not show any difference when only those lying within 150 nm (close to the size of well-formed pits, Fig. 3i**,** yellow arrow) are compared, clearly indicating the population with AP2 at edges start making this difference significant.

Together, the data showed that triggering de-adhesion reduced tension, and cells ramped up the frequency of their endocytosis events. We also know from following the fluctuations that the lowering of tension was also stalled or recovered back at later stages of de-adhesion (Fig. 2b). To understand if endocytosis affected tension regulation and how we next used pharmacological agents to block endocytosis and followed treated cells on de-adhesion.

### Blocking dynamin-dependent pathways reduces de-adhesion-triggered endocytosis

We studied the effect of blocking dynamin-dependent pathways (Fig. 4, Fig. S6, S7) by treating HeLa cells with Dynasore [31]. Specifically, it does not stop the formation of pits (clathrin-coated or caveolae, among others) but prevents dynamin’s function in the scission of pits (Fig. 4a). Through Tf-uptake assay Dynasore’s effect on stalling of pit scission is clear (Fig. S6 e, h, i). Instead of the rise in Rab5’s area fraction on de-adhesion, an initial drop was observed with de-adhesion as expected since early endosomes were expected to contain Rab5 [53]. (Fig. 4b, c, Fig. S7 a, b). Failure of fission caused tubes to form, which also contained Rab5 (arrows, Fig. 4b, Fig. S7a). The increase in Tf puncta in Dynasore-treated cells was enhanced in de-adhering cells (Fig. 4c, d).

Rab4 labelling (immunofluorescence) revealed an increase during the de-adhesion (Fig. 4b, Fig. S7b). This data suggested that the intermittent accumulation could be due to its inability to fuse normally with the plasma membrane (with reduced tension). This aligns with studies that have reported reduced recycling on inhibiting Dynamin [14, 15].

Thus, Dynasore drastically reduced the formation of new early endosomes while also inhibiting the fusion of recycling endosomes with the plasma membrane. Together, these indicate that Dynasore effectively brought down de-adhesion-triggered endocytosis in the cell. We next studied how such blocking of endocytosis would affect the tension regulation during de-adhesion.

### Inactivating dynamin does not stop tension recovery, but knocking down AP2 does

The single cell distribution of fluctuation amplitude and tension clearly changes as de-adhesion progresses to P2, implying that the tension reduced on de-adhesion of Dynasore treated cells (Fig. 4e–f, Fig S7d–i). Interestingly, instead of preventing tension recovery, it was enhanced in Dynasore-treated cells (Fig. 4e) in comparison to untreated cells (Fig. 2d). Normalized cell averages (Fig. 4g, Fig S7 f) point to the augmented mechanical regulation operating from P2 to P3 in Dynasore-treated cells. Maps help us visualize this (Fig. 4f, Fig S7e). On checking the state of activity (entropy generation rate), we found a lowering of activity on Dynasore treatment (Fig. S7g).

To further validate, we performed experiments with a dominant-mutant of dynamin that was transiently transfected in cells. We found that even in these cells, the increase in tension or reduction in SD_time_ was more substantial than in the control condition (Fig. S8). Clearly, the tension at P3 was higher than C, unlike in control sets.

The data imply that although endosome formation is reduced, membrane mechanics regulation during de-adhesion is not stopped. This clearly implies that the formation of pits has a direct role in tension recovery. To verify, we next compared the excess areas from IRM images to find the impact of Dynasore on membrane smoothness. There was a significant reduction in the excess area in Dynasore-treated cells, implying a smoother membrane (Fig. 4g), supporting the hypothesis that pit formation can reduce excess area and enhance the effective tension. As a control, we checked how excess area changed on inhibiting Cdc42 by ML141. Cdc42 is required for the formation and scission of endocytic pits of the CG pathway. Inhibiting it for 30 min enhanced the excess area (Fig. S7h), further supporting the hypothesis that pit-formation reduces excess area.

To validate further the role of pit-formation in tension recovery, we used siRNA-mediated knocked-down AP2. The knock-down was confirmed by immunofluorescence (Fig. S7j) and western blot (Fig. S7k) using control cells and cells treated with either scrambled siRNA (Scramble) or AP2-siRNA (AP2-siRNA). Depletion of AP2 by siRNA has been shown [25] to reduce membrane clathrin as well drastically reduce coated pits on the plasma membrane. We observe that AP2-siRNA significantly reduced tension and enhanced fluctuations and excess area (Fig. 4i top) of cells. During de-adhesion (Fig. 4i bottom), the tension reduced in the P2 phase but failed to be regulated back in the P3 phase (Fig. 4h, i). SD_time_ increased with de-adhesion (Table S2), although when compared using cell-wise statistics, no significant change was noted (Fig. 4i). However, it was clearly not regulated back. The response of AP2-siRNA-treated cells were marked different than control cells or cells treated with scramble siRNA. Together, the Dynasore, dynamin mutant and AP2 siRNA data clearly establish the pit formation step of clathrin-mediated endocytosis to contribute to tension regulation during de-adhesion.

Having provided evidence suggesting pit formation as the critical step in endocytosis to cause tension increase, we next aimed to understand the nature of processes used for the initial pit formation. Dynamin-dependent pathways are not limited to major pathways like CME/caveolae. To understand the dependency on pathways that use key energy-consuming molecules like dynamin or actin, we next check the regulation’s dependency on ATP. ATP-dependent-pit formation is common (CME and caveolae [69], for example). However, multiple pathways, like once induced by Shiga toxins, are ATP-independent [57].

### ATP-depletion does not block tension regulation

We used ATP depletion prior to de-adhesion to investigate the role of active processes and actin polymerization in the mechanical changes observed during de-adhesion (Fig. 5a, b). ATP-depleted cells showed low initial fluctuations but a similar trend of increase in fluctuations followed by a decrease as observed for control cells (Fig. 5a, b, Fig. S9a, Table S1). The effective tension reduced and then increased. The distribution of local values in single cells also captured the changes which were found to be significant. No appreciable change was observed in the area fraction of Rab5 in ATP-depleted cells as de-adhesion progressed (Fig. 5c, d, Fig S9b). The area fraction of RAb5 in ATP-depleted cells was lower than in control cells (Fig. S9b), expected from reduction in active endocytosis.

Therefore, the data here suggest the use of mechanisms that start recovering the tension drop without critically requiring ATP. As the first steps to identify the involved pathway, we next checked the dependence of tension regulation on cholesterol in the ATP-compromised condition. We do so because known pathways (like FEME used to internalize Shiga toxin) using ATP-independent pit formation are cholesterol-dependent and depend on the ability of cholesterol to cluster lipids and initiate the creation of invaginations [33].

### Tension regulation in ATP-depleted cells is cholesterol-dependent

We used ATP depletion and cholesterol depletion as controls (Fig. 6a, S9e) to assess the role of cholesterol in tension regulation in ATP-depleted cells during de-adhesion. Comparing single-cell distributions or cell-averaged values (Fig. 6b–d) showed that while the initial (C-P2) fluctuation (SD_time_) increase displayed the same trend as for the controls, fluctuations from P2 to P3 were not reduced efficiently on combined depletion of ATP and cholesterol. Depleting ATP or cholesterol enhanced the recovery of tension (from Control), but on dual depletion of ATP and cholesterol, there was a further reduction (Fig. 6a, c, Fig S9 c, d) [clear from distributions of local measurements, tension maps (Fig. S9e) and the LMM analysis of the local values (Fig. 6d)].

Together, we showed (Fig. 6e) that the tension drop was significant even on perturbing scission, ATP or cholesterol content and faster in the case of the latter two. The recovery was also significant and faster for these perturbations but could not be effective under reduced ATP and cholesterol-depleted conditions. Treating tension reached by the control cells at the P3 phase as a reference, Dynasore treatment effectively tilted the balance towards a larger tension recovery rate while dual depletion of ATP and cholesterol resulted in lower and, therefore, appreciably slower recovery.

Therefore, the observations strengthen the hypothesis that in the absence of ATP, cells also use cholesterol-dependent processes for mechano-regulation. Since in pathways involved in Shiga-toxin's endocytosis, toxin-rich tubules have been reported to be formed even in ATP-depleted cells/giant unilamellar vesicles [59], we next checked if ATP-depleted cells formed tubules when tension was lowered by de-adhesion. For this, we imaged cells transfected with EGFP-CAAX and quantified the membrane cross-section at higher planes in ATP-depleted and ATP and cholesterol-depleted cells using confocal microscopy.

### On de-adhesion, ATP-depleted cells make more cholesterol-dependent tubular invaginations

Cells were transfected with EGFP-CAAX [41] to label the plasma membrane and taken through different treatments (Fig. 7, S10a). They were subsequently fixed (at 3, 6 min after treatment). Since fixation is not instantaneous, we measured the spread area of cells after fixation to determine the decrease in spread area (Fig. S10b). We observed a reduction to ~ 40% of initial when fixed at 3 min and ~ 17% of initial when fixed at 6 min. Therefore, they were classified as P2 and P3. Cells were subsequently imaged in confocal microscopy at a final resolution of ~ 120 nm in *x* and *y* directions (Fig. 7a, b, S10). From the intensities obtained from scans under the membrane (Fig. 7b, Fig. S10 c, d) along multiple line regions of interest (ROI), each of length ~ 4 µm, peaks were detected with larger widths and heights (from basal intensity) than set thresholds from the line scans (Fig. 7c). After de-adhesion, ATP-depleted cells displayed significantly more internal surface-connected structures than ATP + cholesterol-depleted cells (Fig. 7d) at the P2 and P3 phases. It should be noted that on cholesterol deletion (without ATP depletion, Fig. S10 e) or in control cells (no drug treatment, Fig. S10 f), there were much fewer tubules (Fig. S10 g), whose number also did not appreciably increase on de-adhesion (Fig. 7d, Fig. S10 e–g). The number of peaks transiently increased with de-adhesion in ATP-depleted cells but, in contrast, decreased for cells also depleted of cholesterol (Fig. 7e, Fig. S10). Tubule length also increased from before trypsinization to 3 min post-trypsinization. Hence, we concluded that the ATP-depleted cells might gain more cholesterol-dependent tubules on de-adhesion, thereby causing the tension surge.

In conclusion, we show that de-adhesion induces a tension drop in the whole cell due to an altered adhesion state. The regulation sets in soon and engages endocytic pathways that internalize the membrane but also populate a recycling pool. Tension reduction is stalled but must be balanced by the recycling back of the membrane, because perturbations blocking internalization enhance the tension recovery rate. ATP and cholesterol depletion inhibits tension recovery, and this inhibition cannot be achieved by either preventing internalization or depleting only ATP or cholesterol individually.

## Discussion

Loss or remodelling of the adhesion machinery are known determinants of malignant transformation [42, 47], while regulated de-adhesion has been shown to aid tumour invasion [26]. In this study, we aimed to understand the basic steps of endocytosis-mediated tension regulation during de-adhesion using IRM as the primary tool. It should be noted that tension was derived using a model that does not account for the frequency-dependent activity factor. We have evidenced that membrane fluctuations assayed at such small patches (0.75 μm^2^) has overall weak activity [43] as inferred from measurements of the lower bound of the entropy generation rate. Using the same algorithm [43], change in activity is clear when endocytic activity is blocked by Dynasore (Fig. S7g), indicating that the technique can resolve small changes. The excess area was observed to be reduced, and fluctuation-tension was enhanced (Fig. S7). While incorporating the frequency dependence of activity factor would be ideal while extracting fluctuation-tension, such formulations are currently not yet feasible for easy application to our data.

Hence, direct measurements of fluctuations (SD_time_) are first used to comment on the state of the membrane and the effective fluctuation-tension, excess area and functional state of the membrane (endocytic activity) used for further inferences. We also observed that during de-adhesion, the active nature of fluctuations did not significantly increase (Fig. S3c). SD_time_ supported inferences about fluctuation-tension, while apparent tension—measured by optical trapping experiments—validated the initial tension drop on de-adhesion. Furthermore, we not only reported changes in these parameters but also presented functional evidence of the altered physical state. Lowered tension state correlated with enhanced endocytosis (accumulation of Rab5 near the plasma membrane, Fig. 3g) and reduced recycling (accumulation of Rab4 near the plasma membrane, Fig. 3g). Importantly, we present straightforward evidence of the functional connection between higher fluctuations (and lower effective fluctuation-tension) leading to enhanced endocytosis which in turn leads to reduction of fluctuations. Active models of cell membrane undergoing endocytosis and exocytosis had been used in theoretical work [55] showing that active endo/exocytosis could give rise to an effective membrane tension. Their application to IRM data was not possible, but the pattern of changes of fluctuations (or effective fluctuation-tension) and Rab5/Tf punctas indicate a similar relationship. Future studies measuring the evolution of fluctuations and endocytosis in the same cells would further clarify their local dependence.

Our data first highlight that the local (at the basal membrane) build-up of membrane fluctuations is actin-dependent and not solely dependent on de-adhesion/retraction. The changes in fluctuations were quick to resolve, and tension was negligibly altered in Cyto D-treated cells. While this aligns with recent work implicating the cytoskeleton–membrane connections in delaying tension flow and, therefore, its equilibration, further studies are needed to prove this conclusively.

Identifying the stage when tension recovery was initiated was our primary target of investigations. We believe that the data strongly suggest that the formation of new invaginations starts the tension recovery. Even in the absence of de-adhesion, data in this manuscript (Fig. S7d) and reported earlier show that enhanced pit-formation (Dynasore treatment) could lead to a reduction of fluctuations and increase in tension (Fig. S7) while suppressing pit-formation by knocking down AP2 increases excess area, decreasing tension (Fig. 4i). We have also observed that Shiga toxin created tubules and increased tension of ATP-depleted HeLa cells that contained GB3 but did not either create tubules or enhance tension in HeLa cells lacking GB3 (data not shown). Such tubules have also been reported to passively regulate the area of lipid bilayers on being strained [72]. The reverse, where flattening of invagination helps cells buffer a tension surge, has already been demonstrated. Hence, it is not far-fetched or physically impossible to use invaginations to perform this task. We believe it is possible that clustered lipids/proteins that initiate the pit formation can already start damping the fluctuations, although reports suggest so in simulations [52]. Clustering of resources, as reported for hotspots of CME [35, 50] could also be ideal sites where pit-formation could remove the excess area. Pinching-off of endocytic buds or other ATP-dependent mechanisms, therefore, may not be critically essential for enhancing tension but required to prevent an excessive rise in tension, which could potentially inhibit many membrane processes or start unwanted processes. ATP-dependent machinery, we propose, acts as mechano-stats in the cell. They could be vital for keeping tension surges in check rather than being required only for enhancing tension. Our hypothesis is strengthened by the observation (Fig. 6e) that the rate of tension-lowering is enhanced in cholesterol-depleted cells, which incidentally, also have a higher fraction of membrane covered by actin-membrane linker—Ezrin (Fig. S11). Whether other lipid-raft-dependent mechanisms are also at play [51] or cholesterol-dependent mechanisms downstream of membrane and actin-remodelling cannot be ruled out.

Finally, some conclusions may be drawn about the relevance of the different pathways of endocytosis in tension regulation during de-adhesion. Assuming the three main pathways to be possibly the CG pathway, CME and caveolae-dependent pathway, cholesterol depletion is expected to block the CG and caveolae-dependent pathways even before pit formation. Cholesterol depletion, despite being reported to drastically (~ 80%) reduce internalization of CME, still is expected to allow ~ 50% of deep pits to form [73]. We observe that cholesterol depletion does not abolish tension recovery or its maintenance close to the Control's response (Fig. 6e, C-P3). However, knocking down AP2, an adaptor protein required for the formation of CCPs during constitutive endocytosis, completely impairs the tension recovery. A lower colocalization with AP2 and more edge localization further confirm enhanced pit formation on de-adhesion. Together, this provides compelling evidence that pit-formation for CME significantly contributes to the mechano-regulation in de-adhering HeLa cells. The passive (ATP-independent mechanisms but cholesterol-dependent) pathways may also contribute by the induction of tubulated structures capturing excess membrane. We suggest that spontaneously pre-clustered platforms poised for other functions might tubulate on sudden tension reduction and contribute to the passive arm of the regulation.

**Fig. 8:**
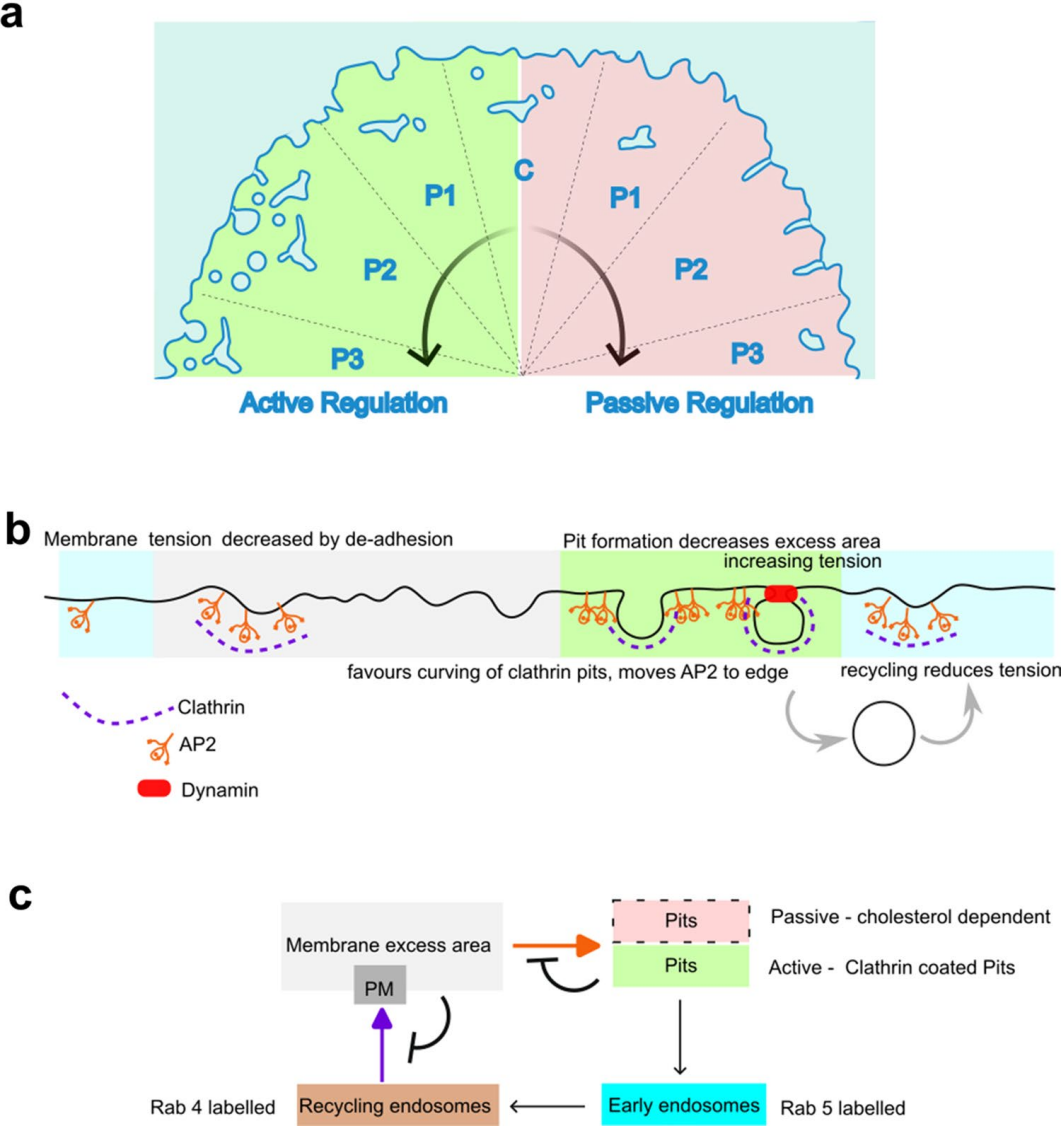
Schematic diagram. **a** Membrane remodelling by active and passive regulation. Schematic shows that membrane fluctuations enhance in the P1 and P2 phase. However, while in passive condition (low ATP), cholesterol-dependent tubules reduce the membrane fluctuations, in normal conditions, active regulation entails formation of pits and their internalization in the P2 phase which transiently accumulate in early and recycling endosomal structures till the tension enhances back and fusion of recycling membrane keeps the increasing tension in check. **b** The decrease in tension by de-adhesion favours curving of clathrin-coated pits—moving AP2 to the edges. Pit formation increases tension. Dynamin-dependent scission and Rab 4-based recycling helps maintain homeostatic tension. Lack of AP2 decreases abrogates regulation by pit formation while lack of functional dynamin affects maintenance of tension and results in much higher tension. **c** Regulation of plasma membrane excess area by formation of pits, early and recycling endosomes. Schematic depicts that higher membrane excess area at the PM favours formation of pits as observed in this study. Such pits can be static or actively internalized to add to the early endosomal pool as shown in this study. Part of the early endosomal pool get converted to the recycling pool which is depleted when some structures fuse back with the PM. At higher membrane excess area, this fusion is disfavoured which can cause accumulation of the recycling endosome as shown in a and observed in this study

In conclusion, in this paper, we have presented the effect of de-adhesion on spatiotemporal fluctuations and effective cell membrane mechanics. We have demonstrated that an initial membrane slack is drastically reduced when the actin cortical network is weak. Pit formation on the plasma membrane has been shown to be a determining factor in regulating tension/fluctuations during the cellular process of de-adhesion. The restoration of the low effective tension used endocytic pit formation for increasing the tension, while the complete regulatory cycle utilized active as well as cholesterol-dependent passive forms of mechano-regulation (Fig. 8).

**Table:** 

Resource availability reagent or resource	Source	Identifier
Pharmacological reagents		
2-Deoxy-D-glucose	Sigma-Aldrich	Cat# D8375; LOT# WXBB7357V
Sodium azide	Sigma-Aldrich	Cat# 438456; LOT# MKBL3422V
Potassium chloride	Sigma-Aldrich	Cat# P9541
EDTA	SRL	Cat# 054448
L-ascorbic acid	Sigma-Aldrich	Cat#A92902; LOT# BCBT6894
L-ascorbic acid sodium salt extrapure	SRL	Cat# 65265
Calcium chloride	Sigma	Cat#C5670; LOT# SLBJ2662V
Dextrose anhydrous purified	Merck	Cat#61780905001730
Sodium chloride	Sigma-Aldrich	Cat# S7653; LOT# 081M0051V
Methyl-ß-cyclodextrin	Sigma-Aldrich	Cat# 332615; LOT# STBH0439
Dynasore hydrate	Sigma-Aldrich	Cat# D7693; LOT# 036M4609V
ML-141	Sigma-Aldrich	Cat# SML0407
Transferrin from human serum, alexa fluor 568 conjugate	Invitrogen	Cat# T2365; LOT# 2136786
Calcein AM	Invitrogen	Cat# C3099; LOT# 2335625
Cytochalasin D	Sigma-Aldrich	Cat# C2618; LOT# 0000084039
Magnesium chloride hexahydrate Cryst. Purified	Merck	Cat# 442615; CAS# 7791-18-6;
Dimethyl sulfoxide	Sigma-Aldrich	Cat# 276855; LOT# SHBC3339V;
HEPES	Sigma-Aldrich	Cat# H3375; LOT# SLBN0452V
Gelatin	Sigma-Aldrich	Cat# G2500; LOT# SLBX2973
Glycine	Merck	Cat# 56-40-6
TritonX-100	Sigma-Aldrich	Cat# 101371902
Recombinant Anti-Rab4 antibody [EPR3043]	Abcam	Cat#ab109009 RRID# AB_10887396
AP2A1 Rabbit mAb	Abclonal	Cat#A4403
Goat Anti -Rabbit IgG, Alexa Fluor 568	Abcam	Cat#ab175471
Goat Anti -Rabbit IgG H&L, Alexa Fluor 488	Abcam	Cat#ab150077 RRID# AB_2630356
Anti-Clathrin heavy chain antibody	Abcam	Cat#ab2731
Cell culture		
Cell line (HeLa)	ATCC	Cat# CCL-2; RRID# CVCL_0030
Dulbecco’s Modified Eagle’s Medium (DMEM, High Glucose)	Gibco	Cat# 11965092
Fetal Bovine Serum, certified, heat-inactivated, US	Gibco	Cat# 10082147
Antibiotic–Antimycotic (100X)	Gibco	Cat# 15240062
TrypLE Express	Gibco	Cat# 12605-028; LOT# 2323753
Trypsin–EDTA (0.05%), phenol red	Gibco	Cat# 25300062; LOT# 2193180
Trypsin–EDTA (0.25%), phenol red	Gibco	Cat# 25200072
Paraformaldehyde	Sigma-Aldrich	Cat# P6148
Phosphate Buffered Saline	Sigma-Aldrich	Cat# P3813
Dulbecco’s Modified Eagle's Medium (DMEM, Phenol Red free)	Gibco	Cat# 21063-029; LOT# 2239707
Opti-MEM (Reduced serum medium)	Gibco	Cat# 31985-070; LOT# 2192861
Lipofectamine 3000 Transfection kit	Invitrogen	Cat# L3000-015; LOT# 2145954
Plasmids		
mCherry- Clathrin LC-15	Addgene	Plasmid# 55019
EGFP-Rab5	Addgene	Plasmid# 49888
mCherry-Rab4a	Addgene	Plasmid# 55125
EGFP-CAAX	Addgene	Plasmid# 86056
AP2 siRNA	IDT	Design id# hs.Ri.AP2A1.13.1
For calibration		
NIST traceable particle size standard, 60 µm	Bangs laboratories	Cat# L130806L; LOT# 11247
Software and algorithms		
ImageJ (FIJI)	NIH	N/A
Origin	OriginLab Corporation	N/A
MATLAB	The Mathworks, Inc	N/A
Labview	National Instruments	

### Lead contact

More detailed information and requests for the resources should be directed to and will be fulfilled by the lead contact, Bidisha Sinha (bidisha.sinha@iiserkol.ac.in).

### Material availability

New materials and methods used in these studies will be available upon request to Bidisha Sinha.

### Data and code availability

Data and codes used in this study for analysis purposes will be available upon request to the lead contact, Bidisha Sinha (bidisha.sinha@iiserkol.ac.in).

## Methods

### Cell line

HeLa cell line (CCL-2, ATCC) was used to perform all the experimental studies.

### Cell culture

HeLa cells were grown in Dulbecco’s Modified Essential Medium (DMEM, Gibco, Life Technologies, USA) with 10% foetal bovine serum (FBS, Gibco) and 1% Anti-Anti (Gibco) at 95% humidity, 5% CO_2_ and 37 °C. Experiments were always performed after 16–18 h of cell seeding.

### Pharmacological treatments

To de-adhere cells from the substrate, HeLa cells were incubated with 0.05% or 0.25% Trypsin–EDTA solution (Gibco) at 37 °C on the onstage microscope incubator. To inhibit all dynamin-dependent endocytic pathways, we incubated cells with Dynasore hydrate (80 μM; Sigma) in serum-free media for 20 min [5, 31, 40]. HeLa cells are incubated with ML-141 (10 μM; Sigma) for 30 min in serum-free media to inhibit CLIC-GEEC endocytic pathways [74]. For ATP depletion, cells are incubated for 1 h with 10 mM Sodium Azide (Sigma-Aldrich) and 10 mM 2- deoxy-D- glucose (Sigma-Aldrich) dissolved in M1 media composed of 150 mM NaCl (Sigma-Aldrich), 1 mM MgCl_2_ (Merck)_,_ 20 mM HEPES (Sigma) [6, 78]. 10 mM Methyl-ß-cyclodextrin (Sigma-Aldrich) was used in serum-free media for 50 min to deplete Cholesterol [7]. For inhibiting Actin filament polymerization, cells were kept in 5 μM Cytochalasin D (Sigma-Aldrich) for 1 h in serum-free media [6]. Cells were de-adhered by TrypLE Express (Gibco). This was used as an alternative to Trypsin–EDTA for de-adhesion experiments [74]. 1 mM EDTA was used for de-adhering cells [34]. All the treatments were incubated at 37 °C inside the incubator. During imaging and de-adhesion, all treatments were maintained at the same concentration. For Calcein AM staining, 2 μM Calcein AM was added in serum free media and incubated for 30. After incubation media was discarded and fresh media was added for the imaging. For endocytosis uptake assay, 10 μg/ml Transferrin Alexa fluor 568 was incubated for 5 min at 37 °C. For stopping endocytosis, HEPES based buffer was used at ice cold temperature. To remove external Tf fluorescence, ascorbate buffer was used at 4 °C [74]. Cells were fixed using ice cold 4% Paraformaldehyde. To follow Transferrin uptake during de-adhesion or in non-de-adhered cells (in normal or dynamin-inhibited conditions), cells were first incubated with 25 nM of Transferrin for 5 min. Subsequently, Transferrin was washed off and de-adhesion was followed. For dynamin inhibition cells were pre-treated with 80 μM Dynasore hydrate and then Transferrin was added.

### Immunostaining

For immunostaining, cells were first fixed with 4% paraformaldehyde (Sigma-Aldrich) for 15 min and subsequently washed twice with phosphate-buffered saline (PBS, Sigma-Aldrich). Next, cells were incubated in 0.1 M glycine (Sigma-Aldrich) for 5 min and then washed again with PBS. Triton-X was used for 2 min and then washed with PBS. For blocking, cells were incubated with 3 ml of 0.2% Gelatin (Sigma-Aldrich) solution for 3 h at room temperature. Primary antibody treatment was done with Recombinant Anti-Rab4 antibody (Abcam) at 1:200 dilution in Gelatin and kept overnight at 4 °C to mark recycling endosomes. Goat Anti -Rabbit IgG H&L, Alexa Fluor 488 secondary antibody (Abcam) was used at 1:500 dilution at Gelatin for 2 h after washing with PBS. Subsequently, cells were imaged in 2 ml of PBS.

### Transfection

EGFP-Rab5 (Addgene) was a gift from Arnab Gupta. Cells were transfected with 1 μg of Rab5 plasmid DNA to label early endosomes, respectively, by using Lipofectamine 3000 (Invitrogen). EGFP-CAAX [41] was a gift from Lei Lu. Cells were transfected with 1 μg EGFP-CAAX (Addgene) plasmid to mark the membrane. Cells were transfected with 1 μg of mCherry- Clathrin LC-15 (Addgene) to label clathrin-coated vesicles. To mark fast recycling endosomes, cells were transfected with 1 μg of mCherry- Rab 4a plasmid. Other treatments, if required, were performed 16 h after transfection.

Fixation of cells was performed by using 4% paraformaldehyde (Sigma-Aldrich) for 15 min at 37°C temperature.

### siRNA-based knockdown

HeLa cells are transfected with 10 nM of AP2 siRNA total for 72 h. At 0 h first siRNA transfection was done and at 48 h fresh media was added with 10 nM of AP2 siRNA booster dose. To validate the knockdown of AP2, cells are immunostained with Anti AP2 antibody to check intensity at the basal membrane by TIRF microscopy. Western blotting was also performed using established protocols to validate the knockdown.

### IRM imaging

Cells were imaged in Nikon Eclipse Ti-E motorized inverted microscope (Nikon, Japan) equipped with adjustable field and aperture diaphragms, 60X Plan Apo (NA 1.22, water immersion) and a 1.5X external magnification on an onstage 37 °C incubator (Tokai Hit, Japan). Either an EMCCD (Evolve 512 Delta, Photometrics, USA) or an s-CMOS camera (ORCA Flash 4.0, Hamamatsu, Japan) was used for imaging. A 100 W mercury arc lamp, an interference filter (546 ± 12 nm) and a 50–50 beam splitter were used [6]. For IRM, movies consisted of 2048 frames (19.91 frames/s, EMCCD and 20 frames/s for s-CMOS) recorded at EM 30 and an exposure time of 50 ms.

### Optical trap experiment

For optical trap-based tether-pulling experiments, a 2 µm polystyrene bead was trapped by focusing a 1064 nm Laser (Coherent, Sweden) of 1000 W power at source using a 100 × objective. The back-aperture of the objective was over filled after beam expansion and using mirrors and a 50/50 beam-splitter for beam manipulation. The trap stiffness (*k*) was calibrated from trajectories of the trapped bead using the equipartition approach $k=\frac{{k}_{B}T}{\langle {x}^{2}\rangle }$ where *x* is the displacement of the bead from the trap centre, *k*_*B*_ is the Boltzmann constant and T the temperature in the Kelvin scale. For analysis, the bead was detected as an object (MATLAB, Fiji), and its centre was tracked with time. The bead’s displacement from the centre of the trap and the spring constant of the trap was used to get the force ($F=-kx$). For every cell, a tether was pulled at a constant velocity of 0.5 µm/s up to a distance of 40 µm (LabVIEW, National Instruments, USA). Tether force was calculated from the average bead position during the period when it was parked with the tether pulled (Fig. S3c) for ~ 50 s. Imaging was done at 200 frames per sec. The apparent membrane tension of the apical section of the cell was derived from the force using the Canham-Helfrich equation ${\sigma }_{A}=\frac{{F}^{2}}{8\kappa {\pi }^{2}}$, where *κ* is the bending rigidity and taken to be 15 k_B_T [13], and σ_A_ denotes the apparent membrane tension.

### TIRF imaging

For TIRF Microscopy, an inverted microscope (Olympus IX-83, Olympus, Japan) was used with a 100X 1.49 NA oil immersion TIRF objective (PlanApo, Olympus). An s-CMOS camera (ORCA Flash 4.0, Hamamatsu, Japan) and 488 nm, as well as 561 nm laser sources, were used. Images were acquired using an exposure time of 300 ms with ~ 70 nm penetration depth.

### Confocal imaging

For confocal imaging, a Leica confocal microscope (Leica SP8) was used with a 63X oil objective lens (NA 1.4). A step size (in z) of 250 nm is used for imaging with a pixel size of 45 nm and deconvoluted (Leica Lightning Software).

### STED imaging

An Abberior Facility Line system with an Olympus IX83 microscope (Abberior Instruments) was used for STED imaging. Abberior autoalignment sample was utilized for alignment of the STED and confocal channels. 15 nm pixel size was maintained during imaging. A pulsed STED line at 775 nm was used for depletion and STAR Red and Alexa 568 conjugated secondary antibodies were used for imaging Clathrin and AP2, respectively.

### Analysis of IRM images

The intensity of IRM images was converted to height (wherever applicable) as reported [6]. The amplitude of spatial undulations spatial (SD_space_) was obtained from the standard deviation (SD) of relative heights across all pixels in an FBR after averaging it over 20 frames. For obtaining the amplitude of temporal fluctuations, SD_time_, the SD of the relative heights over 2048 frames in each pixel was calculated and averaged across all pixels in an FBR. The power spectral density (PSD) of individual pixels was obtained from the temporal relative height time series using either the FFT method or the covariance method (MATLAB). PSDs of all pixels in an FBR were averaged to obtain the PSD for that FBR. For obtaining mechanical parameters, the PSDs were fitted to ${\text{PSD}}\left(f\right)=\frac{4{\eta }_{eff}A{k}_{B}T}{\pi }{\int }_{{q}_{{\text{min}}}}^{{q}_{{\text{max}}}}\frac{dq}{{(4{\eta }_{eff}(2\pi f))}^{2}+{\left[\kappa {q}^{3}+\sigma q+\frac{\gamma }{q}\right]}^{2}}$ [3, 6, 23] where active temperature (A), effective cytoplasmic viscosity (*η*_eff_), confinement (*γ*) and membrane tension (*σ*) were used as fitting parameters. The bending rigidity (*κ*) was fixed at 15 k_B_T [68]. For obtaining excess area fraction ($\frac{\Delta A}{A}=\frac{A-{A}_{P}}{A}$) over an FBR, the flat area of the FBR is taken as A_P_ (= *L*^2^ when patch/FBR is a square of side *L*) and *A* is the sum of all *dA* calculated for each pixel by comparing the height at that pixel with its neighbours ($dA=\sqrt{1+{h}_{x}^{2}+{h}_{y}^{2}}\mathrm{dxdy where} {h}_{x}=\frac{\partial h}{\partial x} and {h}_{y}=\frac{\partial h}{\partial y}$) [16]. The excess area is represented in figures as the percentage excess area ($\frac{\Delta A}{A} \times 100$).

The activity per FBR is calculated as the lower bound of the entropy generation rate at each FBR. For obtaining the entropy generation rate, data pooled from all pixels of the FBR were taken through dimensional reduction using principal component analysis. The time series (2048 frames) of one of the principal components were built and analyzed as described recently [43] using the short-time inference scheme reported earlier [44].

### Tension mapping

For tension mapping (Fig. 2c), PSD was calculated for either for each pixel, and either directly used (pixel-wise tension mapping) or averaged over all pixels in each FBR (FBR-wise tension mapping). PSDs, thus, obtained are fitted and every parameter extracted from fits—including tension and *R*^2^ were mapped on to the same location as the pixel/FBR.

### Fluorescence image analysis

For endosomal or puncta counting (MATLAB), first, a Gaussian blur operation was performed to spatially average out the image. The Gaussian-blur is next subtracted from the original image, thus enhancing local contrast. The subtracted image is normalized between 0 and 1, and thresholding was performed using the appropriate threshold, resulting in a binary image. Next, a mask was applied over the image to select the cell. Single pixels were removed using serial erosion and dilation, and subsequently, the binary image was used to detect objects. The area fraction is calculated by dividing the total area of detected objects ($\sum_{i}{A}_{i}$) by the total area of the ROI or cell (${A}_{ROI}$). Similar approach was used for calculating colocalization. Objects were detected for each channel separately. Total area of the pixels overlapping in the two binary images was considered to be colocalizing area ($\sum_{j}{O}_{j}$). Colocalizing area divided by the total area of the ROI was used as colocalizing area fraction ($\frac{\sum_{j}{O}_{j}}{{A}_{ROI}}$). Colocalizing area divided by object-covered area of any particular was used as the percentage colocalization of that channel ($\frac{\sum_{j}{O}_{j}}{\sum_{i}{A}_{i}) }\times 100$). Mander’s coefficient was obtained using ImageJ.

### Counting tubules from confocal z-slices

For counting tubules, Z-stack images of cells were captured. Line scans were drawn at the cortex regions of cells (ImageJ/Fiji), and the intensities of these line scans were taken from the intensity plot profile of each line scan to plot the internal intensity. Peak analysis (MATLAB) involved evaluating the intensity line scans for peaks of minimum prominence of ~ 6 and width of ~ 5. Peaks were counted as tubules. Length of tubules were obtained by drawing line ROIs along tubules and measuring their length.

### Statistical analysis

Every IRM experiment was preceded by imaging beads. Every experiment was repeated at least thrice involving multiple cells and FBRs (Table S1). A Mann–Whitney *U* test was performed for statistical significance testing (ns denotes *p* > 0.05, *denotes *p* < 0.05, **denoted *p* < 0.001). When indicated, a linear mixed effect model (LMM, MATLAB) was also used (using the fixed effect of time and random effects grouped under replicate set number of the experiments and cell number) to quantify the significance of the observed changes in logarithm of tension values (Table S2). This helped avoid the effect of the high sample size of FBRs that could influence hypothesis testing. LMM has been used for comparisons in other high-sampling mechanical measurements [24, 56].

### Supplementary Information

Below is the link to the electronic supplementary material.

Supplementary file1 (PDF 3906 KB)

## Data Availability

The data that support the findings of this study are available from the corresponding author upon reasonable request. Codes used for this study are available at: https://github.com/BidishaSinha/Mechano-regulation-by-pit-formation-during-De-adhesion.
