# Supplementary material for: Mechano-regulation by clathrin pit-formation and passive cholesterol-dependent tubules during de-adhesion
Source: Cell Mol Life Sci. 2024 Jan 13;81(1):43. doi: 10.1007/s00018-023-05072-4 (PMC10787898; doi:10.1007/s00018-023-05072-4)
Supplement: Supplementary file 1 — Supplementary file1 (PDF 3906 KB) [file 18_2023_5072_MOESM1_ESM.pdf]

Mechano-regulation by clathrin pit-formation and passive cholesterol-dependent tubules during de-adhesion.

Tithi Mandal[1], Arikta Biswas[1+], Tanmoy Ghosh[1], Sreekanth Manikandan[2], Avijit Kundu[3,4], Ayan Banerjee[3], Dhrubaditya Mitra[2], Bidisha Sinha[1]

[1]Department of Biological Sciences, Indian Institute of Science Education and Research Kolkata, Mohanpur, Nadia – 741246, India

[2]NORDITA, KTH Royal Institute of Technology and Stockholm University, Roslagstullsbacken 23, 10691 Stockholm, Sweden

[3]Department of Physical Sciences, Indian Institute of Science Education and Research Kolkata, Mohanpur, Nadia – 741246, India

Email correspondence: bidisha.sinha@iiserkol.ac.in

+Present address: Mechanobiology Institute, National University of Singapore, 5A Engineering Drive, Singapore 117411

[4]Present address: Experimental Physics I, Universität Bayreuth, Universitätsstraße 30, 95447 Bayreuth, Germany

LIST OF FIGURES

Figure S1. Fluctuations and tension of adhered cell - over time

Figure S2. Following the same regions through de-adhesion

Figure S3. Fluctuations during de-adhesion with different strengths of de-adhering agent.

Figure S4. Membrane fluctuation, Tension, and tension maps on Cyto D treatment

Figure S5. *Optical trapping*

Figure S6. Fluorescence analysis, Transferrin, Early and Late Endosome imaging, Clathrin puncta analysis

Figure S7. Early and Late Endosome and Membrane Parameters on blocking endocytosis

Figure S8. Fluctuations and Tension of Dynamin2(K44A)-mCherry transfected cell

Figure S9. Membrane fluctuation, Rab5 area in ATP Depletion, Parameters and tension maps on ATP and cholesterol depletion and both ATP – Cholesterol depletion.

Figure S10. Membrane images marked with EGFP-CAAX

Figure S11. Enhanced surface ezrin on cholesterol depletion

Table S1. List of statistical parameters for all figures

Table S2. Values obtained from LMM analysis of FBR-wise comparisons.

Extended Methods: Details about Linear mixed models.

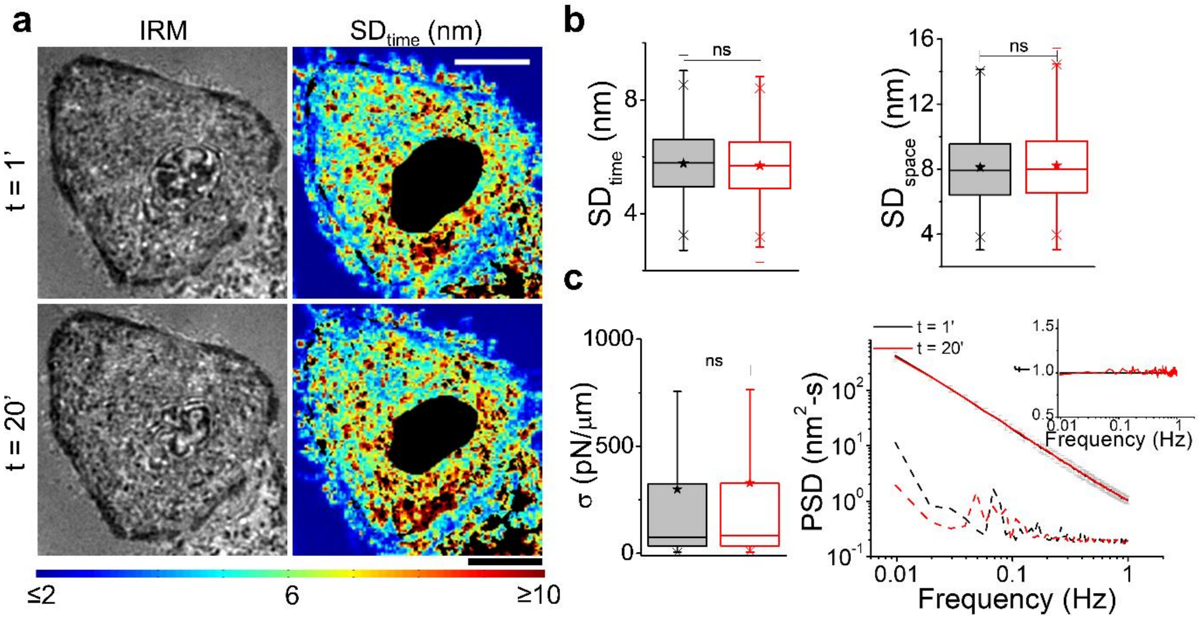

***Figure S1**. **Fluctuations and tension of adhered cell - over time. (a)** Representative IRM and SD_{time} map of adhered HeLa cell imaged at 1 and 20 min. No de-adhesion media was administered. **(b)** Temporal and Spatial fluctuations. **(c)** Tension and power spectral density plot.*

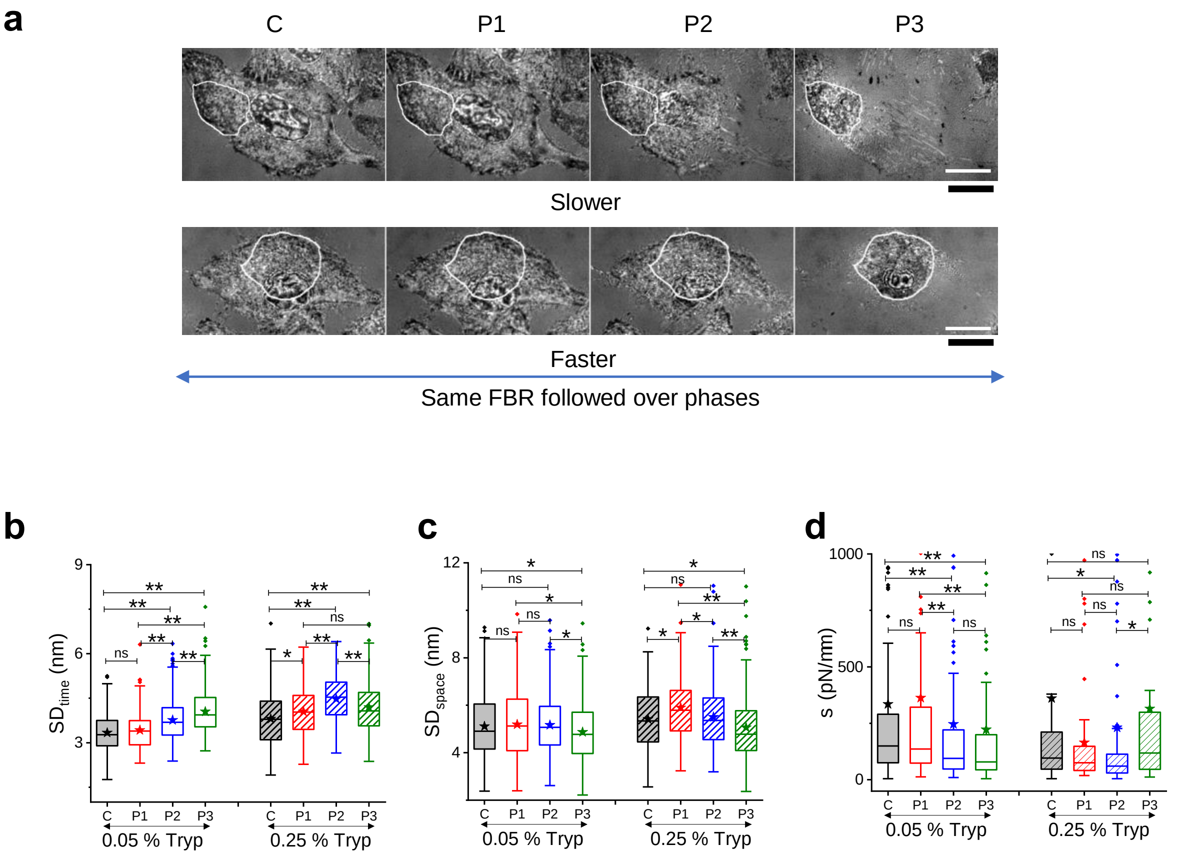

Figure S2. Following the same regions through de-adhesion. (a) *Representative IRM images of HeLa cells during de-adhesion. White outlines mark out the region followed over time.* ***(b)*** *Comparisons of temporal fluctuations and* ***(c)*** *spatial undulations.* ***(d)*** *tension for cells at different phases and for slower (0.05%) and faster (0.25%) de-adhesion.*

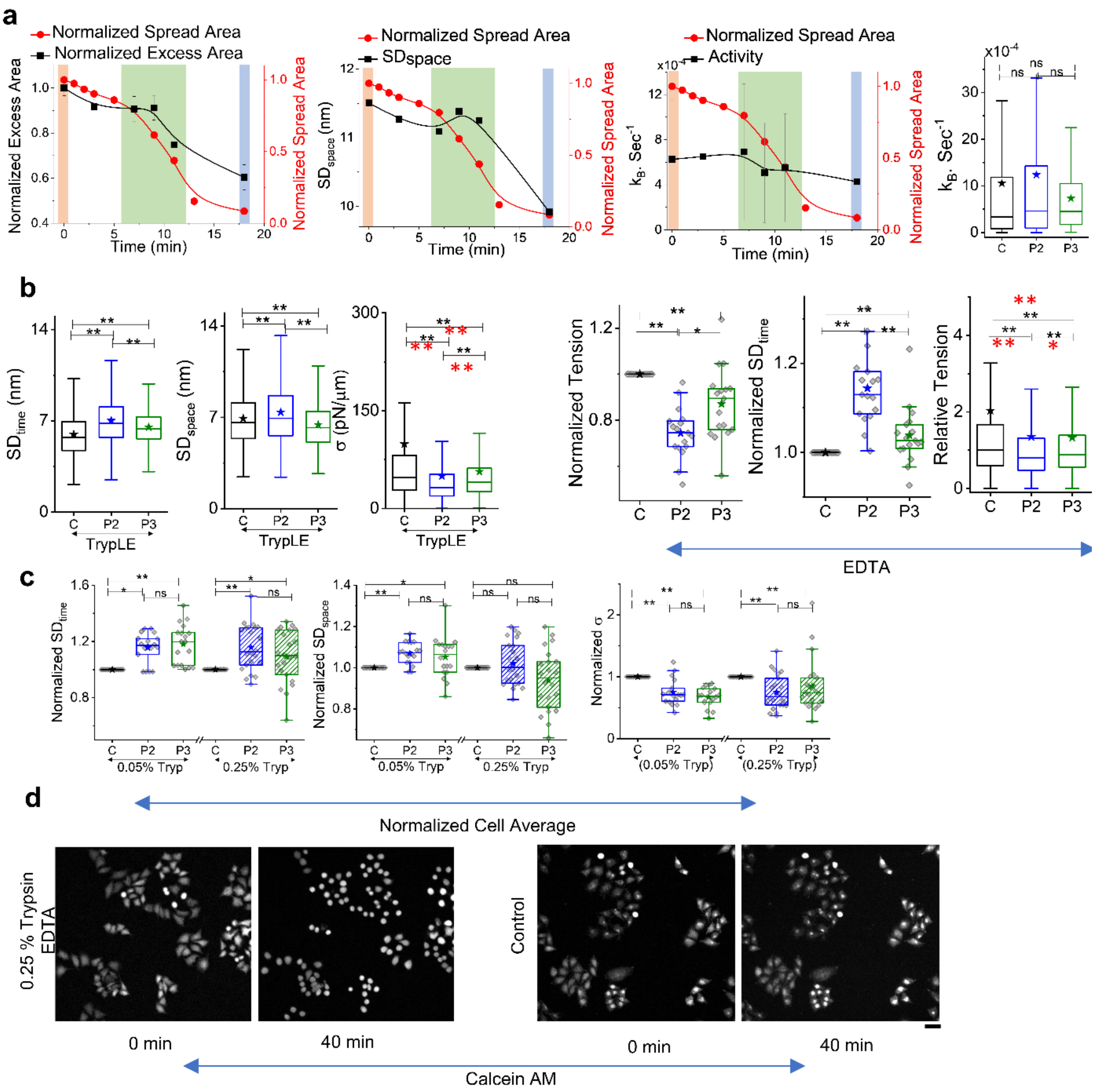

***Figure S3. Fluctuations during de-adhesion with different strengths of de-adhering agent. (a)** Representative time series of indicated parameters for a single cell during fast de-adhesion(left) Box Plot of Activity in different phases of de-adhesion (Right). **(b)** On using milder de-adhesion reagent TrypLE and EDTA. **(c)** Cell-wise comparison of fluctuations and tension. **(d)** De-adhering cells stained with Calcein-AM . Scale bar represents 10 μm.*

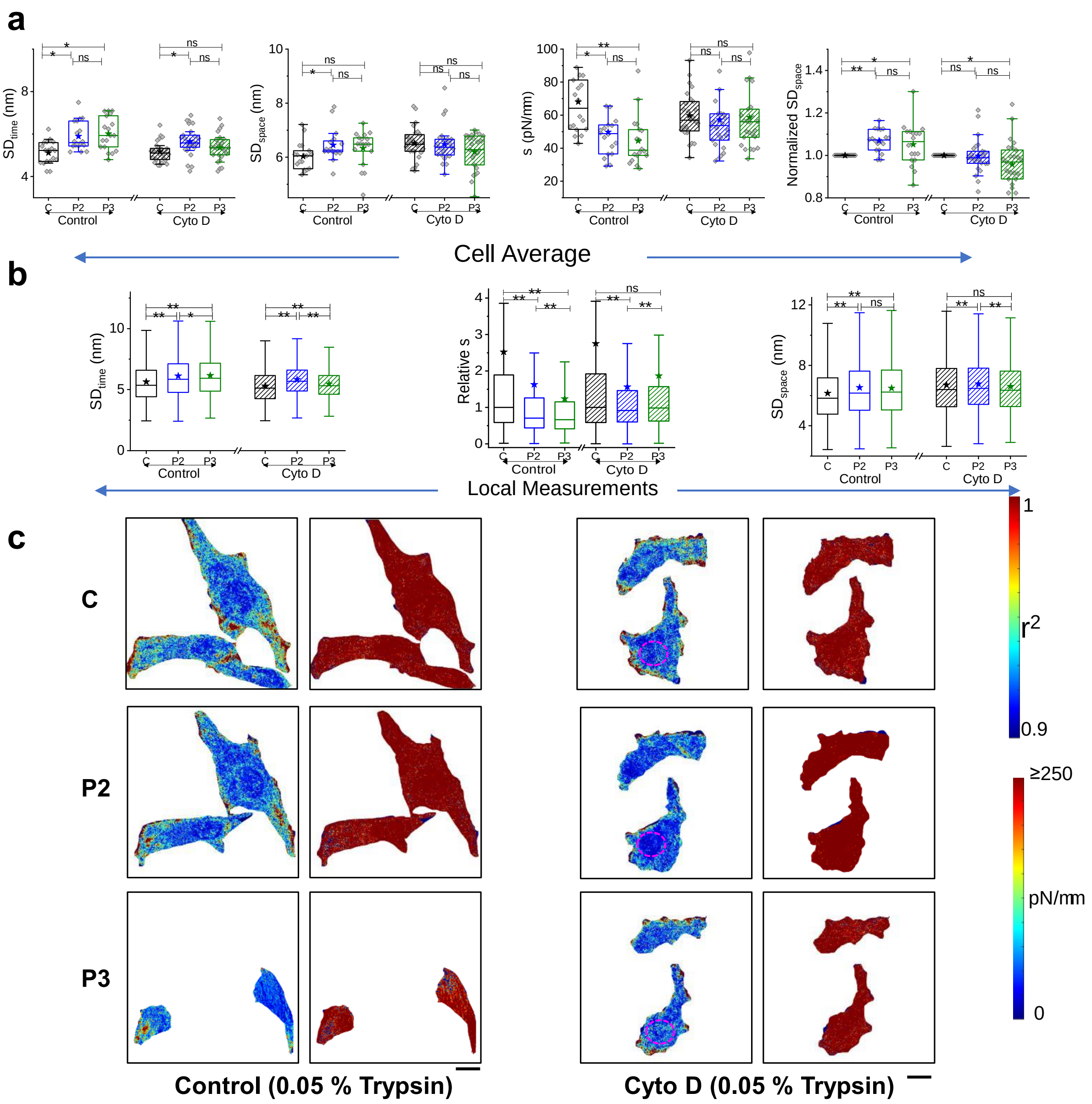

Figure S4. Membrane fluctuation, Tension and tension maps on Cyto D treatment. (a) *Cell-wise comparison of fluctuations and tension.* ***(b)*** *Local measurements of temporal fluctuation, relative tension, spatial undulation at different phases of de-adhesion in control and Cyto D treated cells* ***(c)*** *Typical tension maps of control and Cyto D treated conditions at different phases of deadhesion. Scale bar represents 10 μm.*

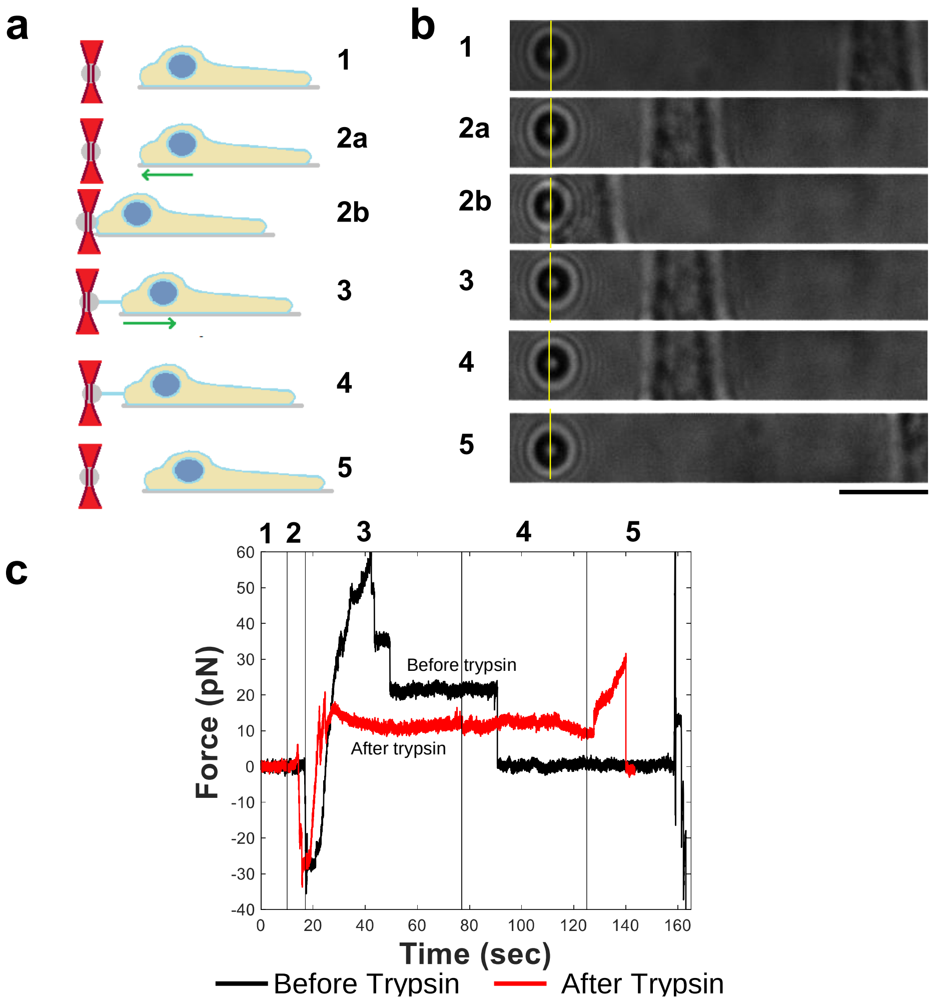

Figure S5. Optical trapping. (a) *Left: Schematic depicts different phases of the optical trapping experiment 1: measuring bead fluctuations away from cell 2a: cell approaching the bead; 2b: Cell and bead interacting; 3: cell pulled away; 4: imaging while bead is parked; 5: moving bead away to rupture the tether to ensure presence of single tether.* ***(b)*** *the brightfield images of the different phases have been put;* ***(c)*** *Force vs. time plot shows the force evolution in the different phases. Note that for the black curve, tether breaks while in the waiting phase and for the red curve in breaks on being pulled again.*

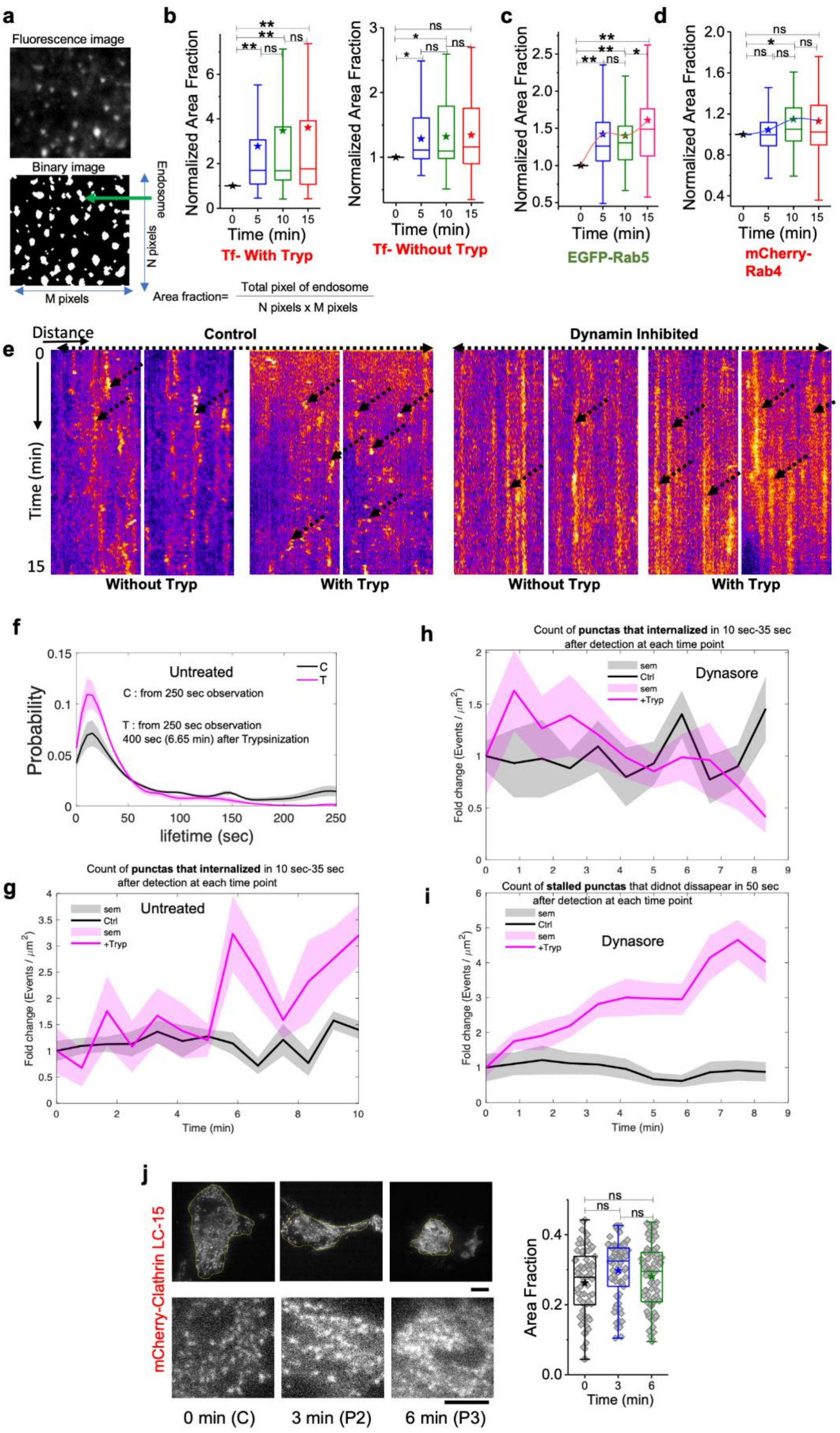

Figure S6. Fluorescence analysis, Transferrin, Early and Late Endosome imaging, Clathrin puncta analysis. (a) Representative fluorescence image and its binary version. Formula depicts how area fraction was calculated. **(b)** *Normalized area fraction (area in μm^2 covered by Transferrin puncta per μm^2) of live cells followed over time as spread area reduces on de-adhesion(left) and without adding de-adhering agent (right).* ***(c)*** *Normalized area fraction of Rab5 of same region followed as the cell de-adheres. Area fraction collated for n=15 regions over n=15 cells.* ***. (d)*** *Normalized area fraction of*

Rab4 of same region followed as the cell de-adheres. ***(e)*** *Kymographs of control and Dynamin inhibited cells in normal and during de-adhesion.* *Arrows point out Transferrin- 568 puncta which remain or disappear after some time.* ***(f)*** *Distribution of lifetime of Tf-punctas analyzed as indicated from ~20 ROIs from 4 cells each of control and trypsinized condition.* ***(g-i)*** *Count of internalized/stuck puncta in control/ dynasore-treated de-adhering cells as indicated.* ***f-i****: TIRF imaging was performed every 5 sec. Objects were detected, kymographs were built for each object (0.745 μm length) and time for kymograph showed presence of object was used as lifetime* ***(j)*** *TIRF images of Clathrin LC-15 transfected cells before and after de-adhesion triggered in the sample. Lower columns are zoomed-in images. Area fraction remain non-significant (in minutes) $n_{cell(Control)(0\ min)}$= 74 cells, $n_{cell(control)(3\ min)}$= 82 cells, $n_{cell(control)(6\ min)}$= 94 cells ,Scale bar= 10 µm.*

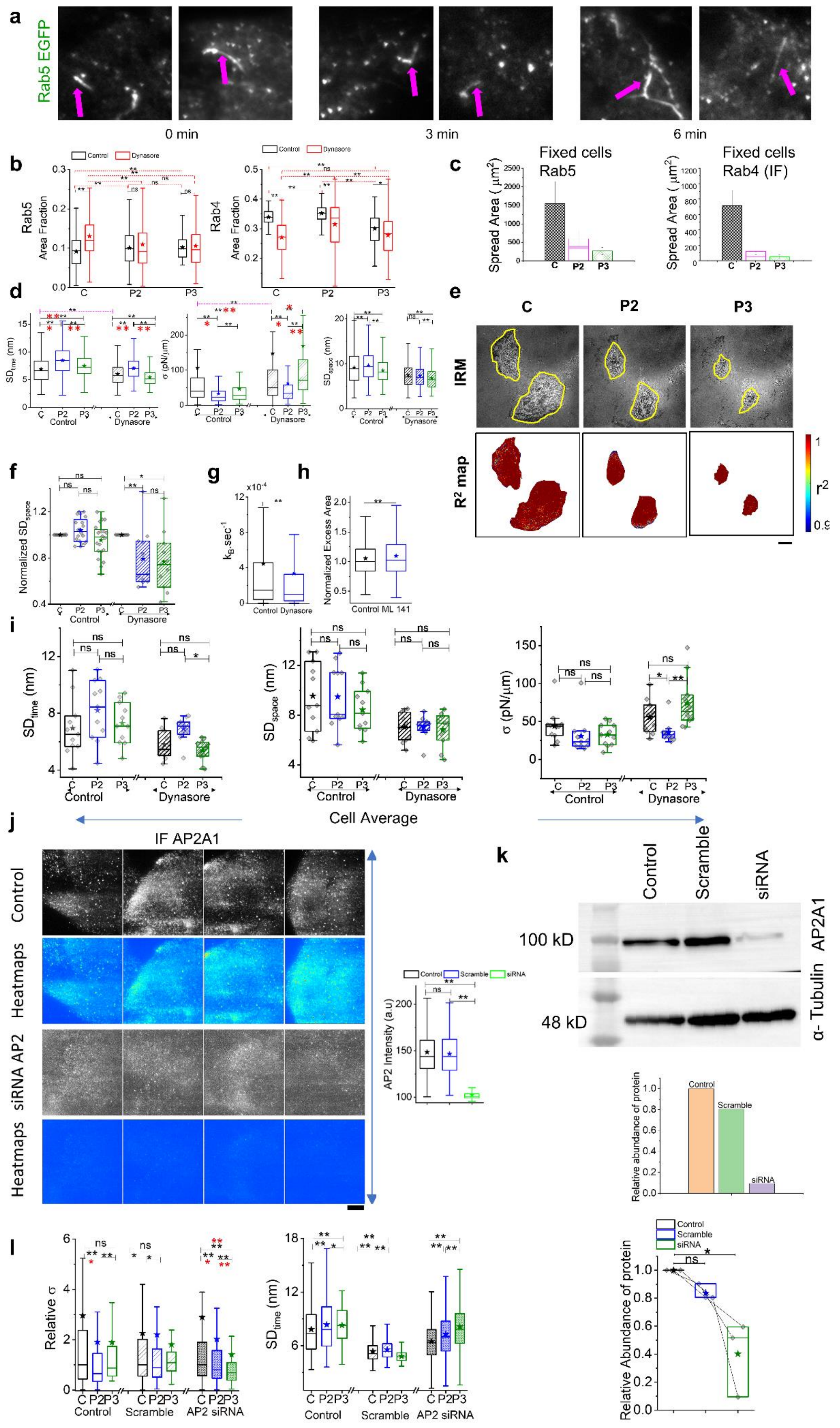
a
Rab5 EGFP
0 min
3 min
6 min
b
c
Fixed cells Rab5
Fixed cells Rab4 (IF)
d
e
C
P2
P3
IRM
R² map
f
g
h
i
Cell Average
j
IF AP2A1
Control
Heatmaps
siRNA AP2
k
Control
Scramble
siRNA
100 kD
48 kD
AP2A1
α- Tubulin
l

Figure S7. Early and Late Endosome and Membrane Parameters on blocking endocytosis. (a) *Representative images of Rab5-EGFP in the first two time points after faster de-adhesion in Dynasore-treated cells. Scale bar represents 10 µm.* ***(b)*** *Comparison between area fraction of control and Dynasore treated Rab5 marked early endosomes (left) and Rab4 marked late endosomes (right).* ***(c)*** *Spread area of control cells transfected with Rab5 (left) and immune-stained with Rab4 before and after addition of 0.25% of trypsin-EDTA.* ***(d)*** *Comparisons made with all FBRs pooled from all cells. n=3 independent experiments. Number of cells=20 (Control), 11 (Dynasore); FBRs of sizes 0.75 μm^2 and 4.67 μm^2 were used for control and 0.75 μm^2 for Dynasore-treated cells.* ***(e)*** *Typical IRM images of Dynasore-treated cells in the different phases of de-adhesion and the corresponding R^2 map.* ***(f)*** *Fold change in spatial fluctuations comparing each cell with its own measurements at different phases.* ***(g)*** *Box plots of activity between Control and Dynasore-treated cells using FBR size of 0.75 μm^2.* ***(h)*** *Box plots of other normalized excess area between Control and ML 141 treated cells using FBR size of 0.75 μm^2.* ***(i)*** *cell-wise comparison of fluctuations and tension. One-way Anova with Bonferroni correction is performed for SD_{time} and SD_{space} since the data is normal. For tension, the Mann Whitney U test is performed and * denotes p value < 0.016 (adjusted by group size of 3 per experiment). n=3 independent experiments. Red * denote LMM was performed.* ***(j)*** *Representative TIRF images of AP2 immune-stained cell under control and siRNA of AP2 treaded conditions. Scale bar = 10 µm. Boxplots of AP2 intensity in control, Scramble and siRNA treated cells. $N_{cell(control)}$ ~82, $N_{cell(Scramble)}$ ~43, $N_{cell(siRNA)}$ ~76.* ***(k)*** *Western blot showing whole cell expression of AP2A1 at control, scramble and siRNA treated conditions and estimation of relative abundance of protein. Number of repeats=3. Cells (control, scramble, siRNA) were scraped in cold PBS and centrifuged at 3500 rpm for 10 min. Cell pellet were lysed by lysis buffer (RIPA+ Protease Inhibitor cocktail+ PMSF) for 1 hr in ice. Lysed cells then sonicated for 5 seconds with 10 min interval for 8 cycle. The whole cell lysate was then centrifuged at 3500 rpm for 10 min at 4° C. Supernatant was then collected and for estimation of protein, Bradford assay was performed. Same amount of protein was taken and ß-mercaptoethanol and NuPage was added to it. Then the mixture was kept at 37˚ C for 30 min. Then this mixture was loaded in SDS PAGE gel (8% of Gel). 0.45 µm PVDF membrane was used for transferring of protein from gel for 55 min at 15 V. After transfer, the membrane was kept in 5 % skimmed milk in TBST for blocking for 3 hours. Primary antibody for AP2A1(α- Adaptin monoclonal antibody 1:1000 dilution) and Alpha -Tubulin (1:20000 dilution) were added and kept at 4˚ C shaker for overnight. After washing with TBS and TBST buffer secondary antibody were added for 2 hours. For secondary antibody HRP tagged Anti Mouse (1: 2000) and HRP tagged Anti Rabbit (1:20000) were used for AP2 and α- Tubulin respectively. Blots were developed by using Clarity Max ECL substrate in the Chemidoc system.* ***(l)*** *FBR wise box plot of temporal fluctuations and tension respectively of Control, Scramble and AP2A1 siRNA treated cells. Red * denote LMM was performed.*

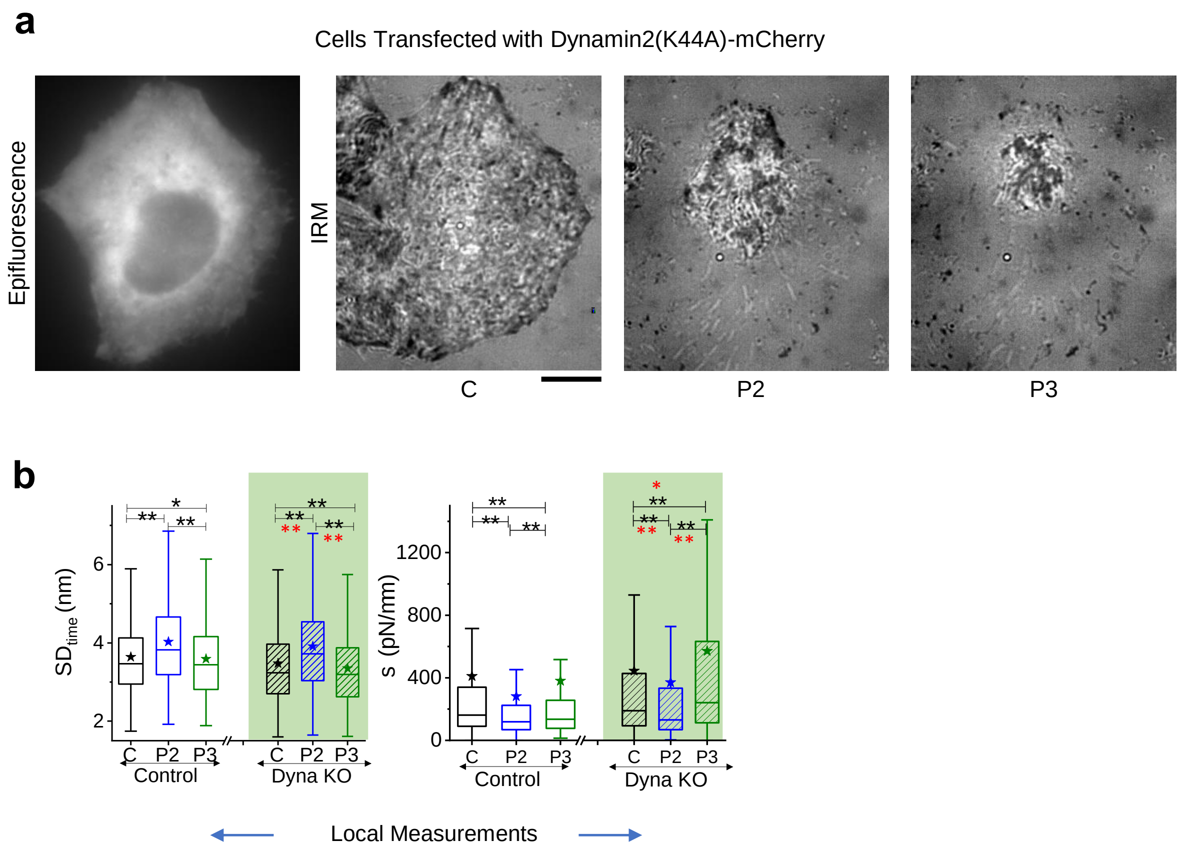

Figure S8. Fluctuations and Tension of Dynamin2(K44A)-mCherry transfected cell (a) *Representative Epifluorescence and IRM images of HeLa cell transfected with Dynamin2(K44A)-mCherry plasmid. Scale bar = 10 μm.* ***(b)*** *FBR wise comparison of temporal fluctuations and tension.* Red * denote LMM was performed.

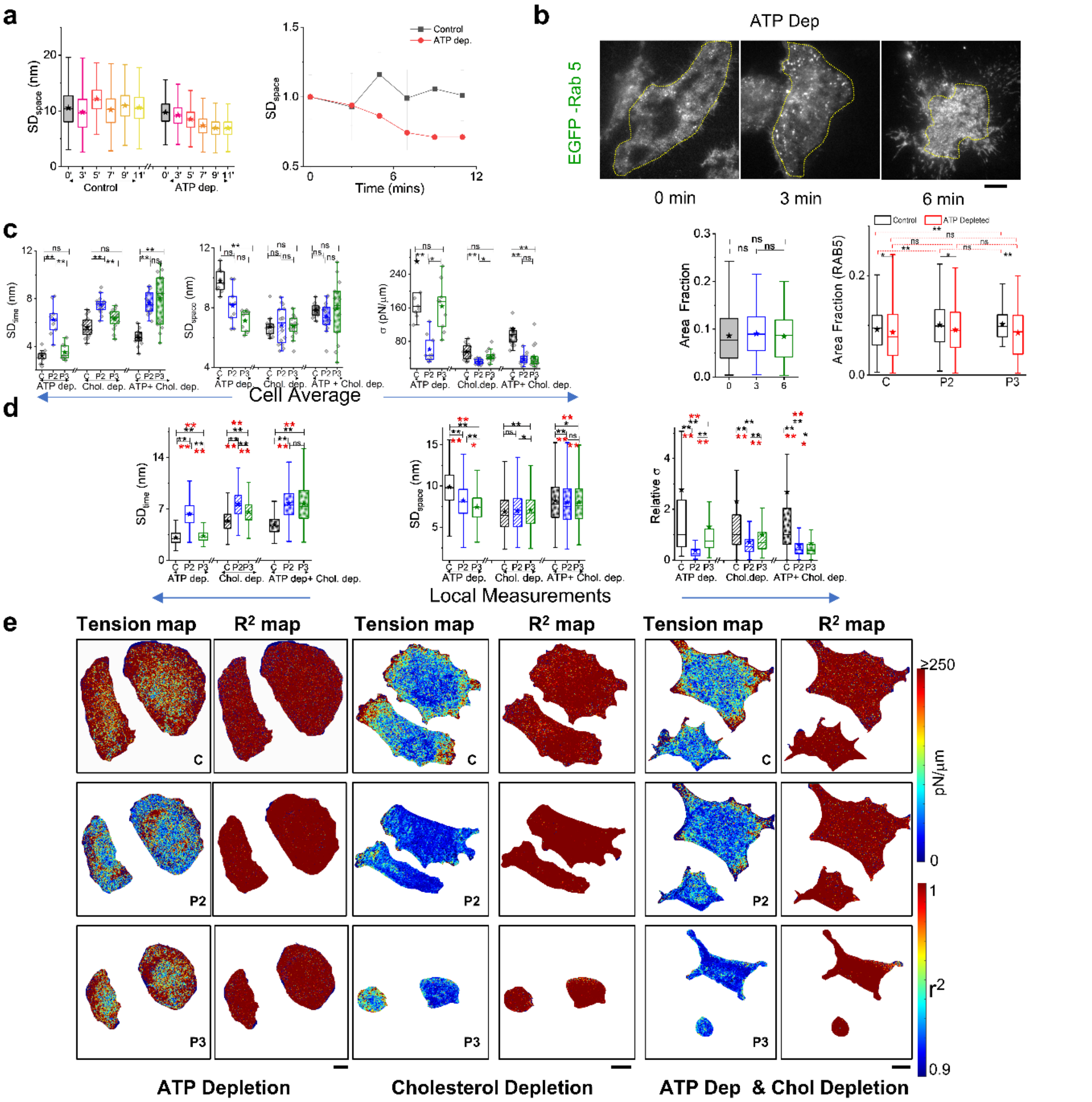

Figure S9. Membrane fluctuation, Rab5 area in ATP Depletion, Parameters and tension maps on ATP and cholesterol depletion and both ATP – Cholesterol depletion. (a) *Time series boxplots and median (with MAD as error bar, lower panel) for different parameters for control and ATP-depleted cells on de-adhesion using an FBR size of 4.67 μm^2.* ***(b)*** *TIRF images of different cells transfected with Rab5 before and after de-adhesion triggered in ATP Depleted cells and Area Fraction of Rab5 of control and ATP Depleted cells.* ***(c)*** *Cell-wise comparison of fluctuations and tension.* ***(d)*** *FBR-wise comparison of fluctuations, relative tension at different phases of de-adhesion in ATP-depleted, cholesterol depleted and ATP-and-cholesterol depleted conditions.* ***(e)*** *Typical tension maps in ATP-depleted, cholesterol depleted and ATP-and-cholesterol depleted conditions at different phases of de-adhesion. Red * denote LMM was performed. Scale bar represents 10 μm.*

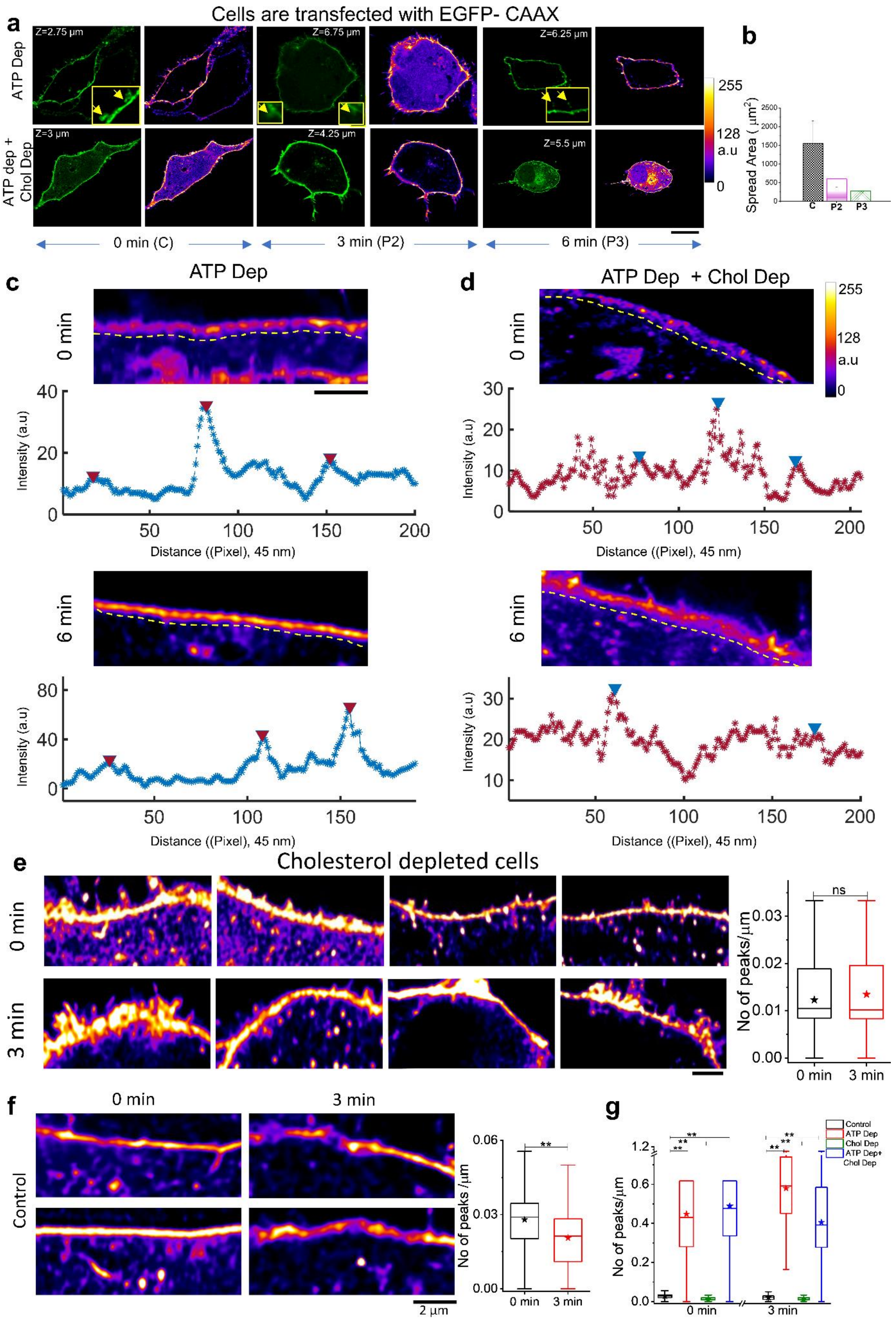

a
Cells are transfected with EGFP- CAAX
ATP Dep
ATP dep + Chol Dep
Z=2.75 μm
Z=6.75 μm
Z=6.25 μm
Z=3 μm
Z=4.25 μm
Z=5.5 μm
255
128
a.u
0
0 min (C)
3 min (P2)
6 min (P3)
b
Spread Area (μm2)
C
P2
P3
c
ATP Dep
0 min
6 min
Intensity (a.u)
Distance ((Pixel), 45 nm)
d
ATP Dep + Chol Dep
0 min
6 min
Intensity (a.u)
Distance ((Pixel), 45 nm)
e
Cholesterol depleted cells
0 min
3 min
No of peaks/μm
ns
f
0 min
3 min
Control
2 μm
No of peaks /μm
g
Control
ATP Dep
Chol Dep
ATP Dep+ Chol Dep
No of peaks/μm

***Figure S10**. **Membrane images marked with EGFP-CAAX.** **(a)** Representative grey scale and colour-coded confocal images of cells (ATP-depleted and ATP Dep+cholesterol-depleted). The rightmost column shows a zoomed-in section highlighting more internal structures in ATP-depleted cells than in ATP Dep +cholesterol-depleted ones. Scale bar = 10 μm. **(b)** Spread area of EGFP-CAAX transfected ATP Depleted cell before and after addition of 0.25% Trypsin- EDTA. Typical images of cell sections showing line ROIs where scans were performed parallel to the membrane in the **(c)** cytosolic side of an ATP depleted cell and **(d)** an ATP Dep +cholesterol-depleted cell at 0 and 6 min after de-adhesion. Scale bar = 2 μm Plots show intensity line scans with triangles pointing out detected peaks with a minimal width and height. Scale bar = 5 μm. **(e)** Representative zoomed-in sections of EGFP-CAAX transfected cholesterol depleted cells and quantification of no. of peaks detected per μm. Scale bar = 2 μm. **(f)** Representative zoomed-in sections of EGFP-CAAX transfected control cells and quantification of no. of peaks detected per μm. Scale bar = 2 μm. **(g)** Quantification of no of peaks in different conditions in 0 min and 3 min.*

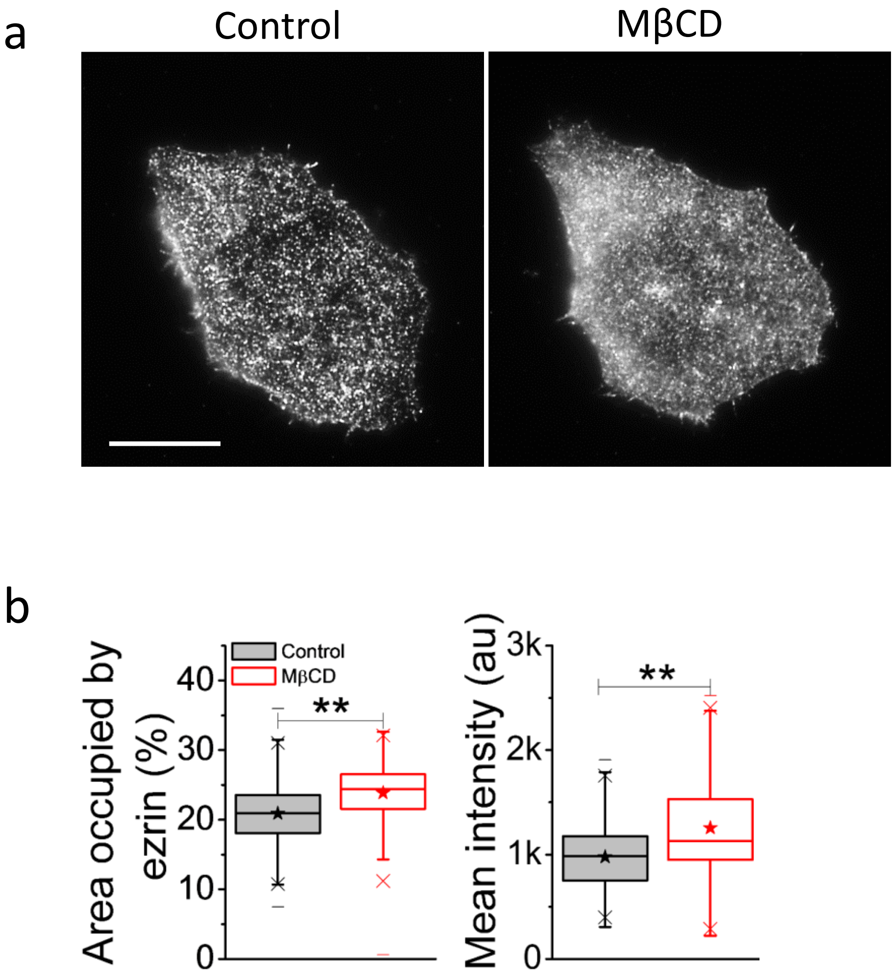

Figure S11. Enhanced surface ezrin on cholesterol depletion. (a) *Representative TIRF images of ezrin immune-stained cell under control and cholesterol-depleted conditions. Scale bar = 10 µm.* ***(b)*** *Boxplots of (left) percentage area and (right) mean intensity occupied by ezrin fluorescent punctas in 3.9 µm x 3.9 µm regions in cells.* N_{cell} *= 15 each.*

Table S1

List of statistical parameters for all figures

Fig 1c											
	**Conditions**	**N_{cell}**		**Mean**	**SD**	**SEM**	**Median**	**p-values (wrt Faster)**			
Time (min)	Control Faster	19		8.87	8.05	2.08	6.20				
	Dyna	7		5.79	4.07	0.74	5.35	0.38			
	Cyto D	10		8.04	2.58	0.91	8.25	3.95E-06			
	ATP Dep	22		1.19	1.10	0.27	0.70	0.84			
	Chol Dep	14		6.06	1.47	0.33	5.75	1.30E-05			
	ATP+ Chol Dep	14		2.20	1.16	0.27	2.05	2.23E-01			
	**Conditions**	**N_{cell}**		**Mean**	**SD**	**SEM**	**Median**	**p-values (wrt slower 0.05% Trypsin)**			
Time	Control Slower	11		12.75	7.15	2.15	10.40				
	Cyto D Slower	18		12.18	10.40	2.45	7.25	0.38			
**Fig 2b**											
**Parameters**	**Conditions**	**N_{cell}**	**n_{FBR}**	**Mean**	**SD**	**SEM**	**Median**	**p-values (wrt C)**	**p-values (wrt P2)**		
Normalized SD_{time}	C	22	~800	1.00	0.00	0.00	1.00		1.17E-04		
	P2	22	~500	1.16	0.17	0.04	1.13	1.17E-04			
	P3	22	~150	1.09	0.19	0.04	1.10	0.03	0.28		
Normalized Tension	C	22	~300	1.00	0.00	0.00	1.00		9.69E-04		
	P2	22	~200	0.75	0.28	0.06	0.68	9.69E-04			
	P3	22	~100	0.85	0.43	0.09	0.74	9.69E-04	0.58		
**Fig 2f**											
**Parameters**	**Conditions**	**N_{cell}**	**n_{FBR}**	**Mean**	**SD**	**SEM**	**Median**	**p-values (wrt C)**	**p-values (wrt P2)**		
Normaliz	C (Control Slow)	18	~700	1	0	0	1		2.47E-03		

ed SD_{time} (Cell-wise)	P2 (Control Slow)	18	~200	1.16	0.11	0.03	1.17	2.50E-03			
	P3 (Control Slow)	18	~470	1.18	0.14	0.03	1.2	<1E-5	0.49614		
	C (Cyto D)	25	~350	1	0	0	1		1.10E-05		
	P2 (Cyto D)	25	~250	1.1	0.12	0.02	1.09	1.10E-05			
	P3 (Cyto D)	25	~700	1.04	0.1	0.02	1.03	7.10E-02	0.06817		
Normalized Tension (Cell-wise)	C (Control Slow)	18	~700	1	0	0	1		2.78E-04		
	P2 (Control Slow)	18	~400	0.75	0.21	0.05	0.71	2.80E-04			
	P3 (Control Slow)	18	~100	0.67	0.16	0.04	0.69	<1E-5	0.33456		
	C (Cyto D)	25	~300	1	0	0	1		0.44		
	P2 (Cyto D)	25	~400	0.96	0.27	0.05	0.92	0.44			
	P3 (Cyto D)	25	~100	0.99	0.27	0.05	0.98	0.07	0.94		

Fig 2g

Parameters	Conditions	N_{cell}	n_{FBR}	Mean	SD	SEM	Median	p-values (wrt Control)			
Force (pN)	Control	10		12.87	4.87	1.54	11.23				
	Trypsin	10		10.73	2.91	0.92	9.9	0.4055			

Fig 3b

Parameters	Conditions	N_{cell}	N_{rois}	Mean	SD	SEM	Median	p-values (wrt control)	
Mean intensity (a.u)	Control	117		4346.27	814.07	75.26	4218.65		
	Trypsin	147		7437.83	1990.15	164.14	7321.15	<0.0001	

Fig 3h

Parameters	Conditions	N_{cell}	N_{rois}	Mean	SD	SEM	Median	p-values (wrt control)	
Manders'	Control	10	27	0.08	0.06	0.01	0.06		
	Trypsin	20	32	0.04	0.03	0.01	0.03	0.007	

coefficient (Clc coloc with AP2)											
**Fig 4g**											
**Parameters**	**Conditions**	**N_{cell}**	**n_{FBR}**	**Mean**	**SD**	**SEM**	**Median**	**p-values (wrt C)**	**p-values (wrt P2)**		
Normalized Tension	C (Control)	20	2006	1.00	0.00	0.00	1.00		5.49E-4		
	P2 (Control)	20	957	0.73	0.28	0.06	0.63	5.49E-4			
	P3 (Control)	20	355	0.83	0.42	0.09	0.72	5.49E-4	0.44		
	C (Dynasore)	11	6669	1.00	0.00	0.00	1.00		0.05		
	P2 (Dynasore)	11	3230	0.73	0.34	0.10	0.65	0.05			
	P3 (Dynasore)	11	1475	1.50	0.76	0.23	1.35	0.05	0.008		
**Fig 4g**											
Normalized SD_{time}	C (Control)	20	3184	1.00	0.00	0.00	6.59		<1E-5		
	P2 (Control)	20	1859	1.18	0.16	0.04	8.44	<1E-5			
	P3 (Control)	20	796	1.10	0.19	0.04	7.23	0.02	0.18		
	C (Dynasore)	11	8861	1.00	0.00	0.00			5.85E-4		
	P2 (Dynasore)	11	4042	1.22	0.24	0.07	6.98	5.85E-4			
	P3 (Dynasore)	11	1733	0.96	0.21	0.06	5.17	0.26	0.01		
**Fig 4g**											
**Parameters**	**Conditions**	**N_{cell}**	**n_{FBR}**	**Mean**	**SD**	**SEM**	**Median**	**p-values (wrt Control)**			
Normalized Excess Area	Control	15	3741	1.07	0.42	0.01	1				
	Dynasore	11	6669	0.99	0.35	0.00	0.9	0			

Fig 4i											
**Parameters**	**Conditions**	**N_{cell}**	**n_{FBR}**	**Mean**	**SD**	**SEM**	**Median**	**p-values (wrt Control)**	**p-values (wrt Scramble)**		
Tension (pN/µm)	Control	18		66.06	31.07	7.32	54.51		0.615		
	Scramble	22		62.00	35.08	7.48	50.90	0.615			
	AP2 siRNA	21		41.99	16.33	3.56	43.93	0.005	0.01		
SD_{time} (nm)	Control	18		5.26	1.07	0.25	5.50		0.82		
	Scramble	22		5.46	0.91	0.19	5.46	0.82			
	AP2 siRNA	21		6.43	1.66	0.36	5.97	0.02	0.02		
**Parameters**	**Conditions**	**N_{cell}**		**Mean**	**SD**	**SEM**	**Median**	**p-values (wrt C)**	**p-values (wrt P2)**		
Normalized SD_{time} (Cell-wise)	C(Control)	9		1.00	0.00	0.00	1.00		0.04		
	P2(Control)	9		1.04	0.08	0.03	1.02	0.04			
	P3 (Control)	9		0.98	0.10	0.03	1.01	0.22	0.29		
	C (Scramble)	5		1.00	0.00	0.00	1.00		0.12		
	P2(Scramble)	5		1.04	0.07	0.03	1.03	0.12			
	P3(Scramble)	5		0.94	0.08	0.04	0.92	0.66	0.09		
	C (AP2)	14		1	0	0	1		0.17		
	P2(AP2)	14		1.09	0.14752	0.03943	1.02	0.17			
	P3 (AP2)	14		1.16	0.22	0.06	1.09	0.04	0.42		
Normalized Tension (cell wise)	C(Control)	9		1.00	0.00	0.00	1.00		0.003		
	P2(Control)	9		0.86	0.14	0.05	0.85	0.003	0.00		
	P3 (Control)	9		0.94	0.16	0.05	0.94	0.003	0.16		
	C (Scramble)	5		1.00	0.00	0.00	1.00		0.12		
	P2(Scramble)	5		0.87	0.19	0.09	0.54	0.12			
	P3(Scramble)	5		1.02	0.20	0.09	0.84	0.66	0.68		
	C (AP2)	14		1	0	0	1		6.42E-4		

	P2(AP2)	14		0.89	0.13	0.03	0.89	6.42E-4			
	P3 (AP2)	14		0.74	0.25	0.7	0.	4.12E-5	0.02		
**Parameters**	**Conditions**	**N_{cell}**	**n_{FBR}**	**Mean**	**SD**	**SEM**	**Median**	**p-values (wrt Control)**			
Normalized Excess Area	Control	15	2278	1.11	0.62	0.01	1				
	Scramble	15	13722	1.30	0.43	0.00	1.23	<0.0001			
	AP2	10	5351	2.17	1.25	0.02	2.04	0			

**Fig 5c**											
**Parameters**	**Conditions**	**N_{cell}**	**$n_{patches}$**	**Mean**	**SD**	**SEM**	**Median**	**p-value (wrt 0 min)**	**p-value (wrt 3 min)**		
Area Fraction Rab 5(ATP Dep)	0 min	26	168	0.09	0.06	0.005	0.08		0.07		
	3 min	41	288	0.09	0.05	0.003	0.09	0.07			
	6min	39	255	0.08	0.05	0.003	0.09	0.52	0.17		
**Fig 6c**											
**Parameters**	**Conditions**	**N_{cell}**	**n_{FBR}**	**Mean**	**SD**	**SEM**	**Median**	**p-values (wrt C)**	**p-values (wrt P2)**		
Normalized SD_{time} (Cell-wise)	C (Control)	11		1.00	0.00	0.00	1.00		0.04		
	P2 (control)	11		1.13	0.17	0.05	1.12	0.04			
	P3 (control)	11		1.06	0.23	0.07	1.06	0.26	0.47		
	C (ATP Dep.)	9		1	0	0	1		1.61E-04		
	P2 (ATP Dep.)	9		1.99	0.46	0.15	2.08	1.60E-04			
	P3 (ATP Dep.)	9		1.11	0.23	0.08	1.19	0.22	0.002		
	C (Chol. Dep.)	16		1	0	0	1		<1E-5		
	P2 (Chol. Dep.)	16		1.36	0.19	0.05	1.33	<1E-5			
	P3 (Chol. Dep.)	16		1.14	0.19	0.05	1.11	0.001	0.002		
	C (ATP Dep. +Chol. Dep.)	15		1	0	0	1		<1E-5		
	P2 (ATP Dep. +Chol. Dep.)	15		1.63	0.27	0.07	1.53	<1E-5			
	P3 (ATP Dep. +Chol. Dep.)	15		1.69	0.43	0.11	1.75	1.70E-05	0.45		

Normalized Tension (Cell-wise)	C (Control)	11		1.00	0.00	0.00	1.00		0.04		
	P2 (control)	11		0.89	0.26	0.08	0.83	0.04			
	P3 (control)	11		0.91	0.37	0.11	0.87	0.05	0.95		
	C (ATP Dep.)	9		1	0	0	1		1.61E-04		
	P2 (ATP Dep.)	9		0.29	0.21	0.07	0.23	1.60E-04			
	P3 (ATP Dep.)	9		0.79	0.35	0.12	0.83	0.22	0.002		
	C (Chol. Dep.)	16		1	0	0	1		<1E-5		
	P2 (Chol. Dep.)	16		0.62	0.15	0.04	0.59	<1E-5			
	P3 (Chol. Dep.)	16		0.85	0.25	0.06	0.87	0.05	0.006		
	C (ATP Dep. +Chol. Dep.)	15		1	0	0	1		<1E-5		
	P2 (ATP Dep. +Chol. Dep.)	15		0.39	0.15	0.04	0.37	<1E-5			
	P3 (ATP Dep. +Chol. Dep.)	15		0.44	0.37	0.09	0.34	1.71E-05	0.65		

**Fig 6e**											
**Parameters**	**Conditions**	**N_{cell}**	**n_{FBR}**	**Mean**	**SD**	**SEM**	**Median**	**p-values (wrt Normal Faster)**	**Signification from 0.25%**		
	Normal Faster (0.25%)	22		-0.03	0.08	0.02	-0.05		0.06		
C to P2 (Cell-wise)	Dynasore	11		-0.05	0.06	0.02	-0.07	0.83	1.41E-02		
	ATP Dep	9		-0.12	0.06	0.02	-0.11	0.00	4.14E-04		
	Chol Dep	16		-0.21	0.11	0.03	-0.18	9.35E-07	<1E-5		
	ATP+Chol Dep	15		-0.18	0.07	0.02	-0.18	4.37E-06	<1E-5		
P3 to P2 (Cell-wise)	Normal Faster(0.25%)	22		0.05	0.16	0.03	0.03		0.15		
	Dynasore	11		0.17	0.15	0.05	0.14	0.02	0		
	ATP Dep	9		0.15	0.09	0.03	0.15	0.01	9.12E-04		
	Chol Dep	16		0.12	0.1	0.03	0.12	0.01	2.53E-04		
	ATP+Chol	15		0.02	0.21	0.05	-0.03	0.09	0.71		

	Dep										
P3 to C (Cell-wise)	Normal Faster(0.25%)	22		-0.01	0.06	0.01		-0.02	0.50		
	Dynasore	11		0.04	0.05	0.02	0.03	0.01	0.04		
	ATP Dep	9		-0.01	0.02	0.01	-0.01	0.53	0.11		
	Chol Dep	16		-0.02	0.04	0.01	-0.03	0.94	0.07		
	ATP+Chol Dep	15		-0.07	0.06	0.02	-0.07	2.23E-05	5.82E-04		

**Fig 7d**											
**Parameters**	**Conditions**	**N_{cell}**	**n_{ROI}**	**Mean**	**SD**	**SEM**	**Median**	**p-values (wrt 0 min)**	**p-values (wrt 3 min)**		
No of tubules (μm^{-1})	ATP Dep (0 min)	20	139	0.45	0.25	0.02	0.43		<1E-5		
	ATP Dep (3 min)	18	124	0.58	0.25	0.02	0.59	<1E-5			
	ATP Dep (6 min)	28	240	0.49	0.23	0.01	0.47	0.14	3.61E-05		
	ATP +Chol Depletion (0 min)	21	149	0.49	0.21	0.02	0.48		9.46E-03		
	ATP +Chol Depletion (3 min)	24	161	0.41	0.27	0.02	0.39	9.46E-03			
	ATP +Chol Depletion (6 min)	21	80	0.41	0.21	0.02	0.38	0.004	6.91E-01		

**Parameters**	**Conditions**	**N_{cell}**	**n_{ROI}**	**Mean**	**SD**	**SEM**	**Median**	**p-values (wrt 0 min)**			
No of tubules (μm^{-1})	Chol Dep (0 min)	8	97	0.01	0.01	0.001	0.01				
	Chol Dep (3 min)	9	113	0.01	0.01	9.93 E-4	0.01	0.85			
**Fig 7e**											
**Parameters**	**Conditions**	**N_{cell}**	**n_{ROI}**	**Mean**	**SD**	**SEM**	**Median**	**p-values (wrt 0 min)**	**p-values (wrt 3 min)**		
Intensity	ATP Dep (0 min)	20	16108	16.91	10.56	0.08	14.56		0		

(a.u)	ATP Dep (3 min)	18	18586	35.89	17.51	0.13	32.06	0			
	ATP Dep (6 min)	28	28062	26.06	15.52	0.09	22.96	0	0		
	ATP +Chol Depletion (0 min)	21	15301	11.68	8.67	0.07	10		0		
	ATP +Chol Depletion (3 min)	24	5166	19.72	6.68	0.09	19.68	0			
	ATP +Chol Depletion (6 min)	21	11615	17.40	9.31	0.09	16.34	0	0		

**Fig 7f**											
**Parameters**	**Conditions**	**N_{cell}**	**N_{tubule}**	**Mean**	**SD**	**SEM**	**Median**	**p-values (wrt ATP dep)**			
Tubule length(µm)	ATP dep	18	68	1.27	0.53	0.04	1.24				
	ATP dep+ chol dep	15	58	0.49	0.18	0.02	0.48	<0.0001			

**Supplementary Tables**

**Fig S1b**											
**Parameters**	**Conditions**	**N_{cell}**	**n_{FBR}**	**Mean**	**SD**	**SEM**	**Median**	**p-values (wrt 1min)**			
SD_{time}(nm)	1 min	5	1102	5.8	1.2	0.04	5.8				
	20 min	5	1207	5.7	1.2	0.03	5.7	0.14			
SD_{space} (nm)	1 min	5	1102	8.1	2.3	0.07	7.9				
	20 min	5	1207	8.2	2.3	0.07	8	0.26			
**Fig S1c**											
σ	1 min	5	444	299.15	534.45	25.36	73.67				
	20 min	5	519	328.58	600.38	26.35	83.01	0.48			

**Fig S2b**										
**Parameters**	**Conditions**	**N_{cell}**	**n_{FBR}**	**Mean**	**SD**	**SEM**	**Median**	**p-values (wrt C)**	**p-values (wrt P1)**	**p-values (wrt P2)**

SD time (nm)	C (Slower)	6	287	3.34	0.66	0.04	3.27		0.14	$<10^{-5}$	
	P1 (Slower)	6	287	3.4	0.62	0.04	3.39	0.14		$<10^{-5}$	
	P2 (Slower)	6	287	3.76	0.74	0.04	3.69	$<10^{-5}$	$<10^{-5}$		
	P3 (Slower)	6	287	4.05	0.76	0.04	3.94	$<10^{-5}$	$<10^{-5}$	$<10^{-5}$	
	C (Faster)	5	155	3.81	0.95	0.08	3.79			$<10^{-5}$	
	P1 (Faster)	5	155	4.05	0.81	0.07	4.03	0.013	0.013	$<10^{-5}$	
	P2 (Faster)	5	155	4.47	0.76	0.06	4.54	$<10^{-5}$	$<10^{-5}$		
	P3 (Faster)	5	155	4.19	0.87	0.07	4.07	4.00E-04	0.22	6.00E-04	

**Fig S2c**											
SD_{space} (nm)	C (Slower)	6	287	5.11	1.35	0.07	4.91		0.49	0.45	
	P1 (Slower)	6	287	5.19	1.41	0.08	5.12	0.49		0.93	
	P2 (Slower)	6	287	5.17	1.25	0.07	5.07	0.45	0.93		
	P3 (Slower)	6	287	4.87	1.22	0.06	4.77	3.00E-02	4.00E-03	2.00E-03	
	C (Faster)	5	155	5.41	1.3	0.1	5.34		0.004	0.76	
	P1 (Faster)	5	155	5.89	1.37	0.11	5.79	0.004		9.00E-03	
	P2 (Faster)	5	155	5.5	1.34	0.11	5.36	0.76	9.00E-03		
	P3 (Faster)	5	155	5.06	1.52	0.12	4.78	0.003	$<10^{-5}$	7.00E-04	
**Fig S2d**											
**Parameter**	**Conditions**	**N_{cell}**	**n_{FBR}**	**Mean**	**SD**	**SEM**	**Median**	**p-values (wrt C)**	**p-values (wrt P1)**	**p-values (wrt P2)**	
Tension (pN/µm)	C (Slower)	6	184	335.18	509.56	37.57	149.47		0.86	1.00E-04	
	P1 (Slower)	6	202	362.72	594.72	41.84	136.41	0.86		3.00E-04	
	P2 (Slower)	6	186	246.62	418.89	30.71	95.09	1.00E-04	3.00E-04		
	P3 (Slower)	6	189	222.29	388.63	28.27	78.86	$<10^{-5}$	$<10^{-5}$	0.16	
	C (Faster)	5	75	360.65	672.46	77.65	96.22		0.17	0.01	
	P1 (Faster)	5	72	164.65	256.43	30.22	75.81	0.17		0.13	
	P2 (Faster)	5	73	231	380.72	44.56	60.85	0.01	0.13		
	P3	5	88	314.	499.	53.2	118.	0.7	0.08	0.01	

	(Faster)			63	88	9	08				

Fig S3b											
**Parameters**	**Conditions**	**N_{cell}**	**n_{FBR}**	**Mean**	**SD**	**SEM**	**Median**	**p-values (wrt C)**	**p-values (wrt P2)**		
SD_{time} (nm)	C (TrypLE)	24	19377	5.96	1.72	0.01	5.73		0		
	P2 (TrypLE)	24	10561	7.02	1.87	0.02	6.8	0			
	P3 (TrypLE))	24	2819	6.52	1.36	0.03	6.37	$<10^{-5}$	0		
SD_{space} (nm)	C (TrypLE)	24	19377	6.91	2.18	0.02	6.6		$<10^{-5}$		
	P2 (TrypLE)	24	10561	7.39	2.51	0.02	6.93	$<10^{-5}$			
	P3 (TrypLE)	24	2819	6.44	1.82	0.03	6.21	0	0		
Ten. (pN/µm)	C (TrypLE)	24	14038	98.88	242.49	2.05	47.25		0		
	P2 (TrypLE)	24	6717	49.87	109.27	1.33	32.28	0			
	P3 (TrypLE))	24	1178	56.57	84.22	2.45	40.38	$<10^{-5}$	$<10^{-5}$		
**Parameters**	**Parameters**	**N_{cell}**	**n_{FBR}**	**Mean**	**SD**	**SEM**	**Median**	**p-values (wrt C)**	**p-values (wrt P2)**		
Normalized Tension	C (EDTA)	17	8921	1	0	0	1		<0.0001		
	P2(EDTA)	17	6074	0.74	0.11	0.03	0.74	<0.0001			
	P3 (EDTA)	17	3579	0.87	0.16	0.04	0.89	0.0006	0.02		
Normalized SD_{time}	C (EDTA)	17		1	0	0	1		<0.0001		
	P2(EDTA)	17		1.14	0.08	0.02	1.13	<0.0001			
	P3 (EDTA)	17		1.03	0.07	0.02	1.03	0.0006	0.0003		
Relative Tension	C (EDTA)	17	8921	2.03	5.15	0.05	1		<0.0001		
	P2(EDTA)	17	6074	1.35	3.30	0.04	0.79	<0.0001			
	P3 (EDTA)	17	3579	1.32	2.28	0.04	0.88	<0.0001	<0.0001		
**Fig S3c**											
**Paramete**	**Conditions**	**N_{cell}**	**n_{FBR}**	**Mean**	**SD**	**SEM**	**Median**	**p-values (wrt C)**	**p-values (wrt P2)**		

**rs**											
	C (Slower)	18		1	0	0	1		0.002		
Normalized SD_{time}	P2 (Slower)	18		1.16	0.11	0.03	1.17	0.002			
	P3 (Slower)	18		1.18	0.14	0.03	1.2	<1E-5	0.5		
	C (Faster)	22		1.00	0.00	0.00	1		1.17E-04		
	P2 (Faster)	22		1.16	0.17	0.04	1.13	1.17E-04			
	P3 (Faster)	22		1.09	0.19	0.04	1.10	0.03	0.28		
Normalized Sd_{space}	C (Slower)	18		1	0	0	1		2.18E-05		
	P2 (Slower)	18		1.07	0.05	0.01	1.07	2.18E-05			
	P3 (Slower)	18		1.05	0.1	0.02	1.06	0.02	0.49614		
	C (Faster)	22		1	0	0	1		1		
	P2 (Faster)	22		1.02	0.11	0.02	1.00	1			
	P3 (Faster)	22		0.94	0.15	0.03	0.95	0.10	0.06		
Normalized Tension	C (Slower)	18		1.00	0.00	0.00	1.00		2.78E-04		
	P2 (Slower)	18		0.75	0.21	0.05	0.71	2.78E-04			
	P3 (Slower)	18		0.67	0.16	0.04	0.69	<1E-5	0.33		
	C (Faster)	22		1.00	0.00	0.00	1.00		9.69E-04		
	P2 (Faster)	22		0.75	0.28	0.06	0.68	9.69E-04			
	P3 (Faster)	22		0.85	0.43	0.09	0.74	9.69E-04	0.58122		

**Fig S4a**										
**Parameters**	**Conditions**	**N_{cell}**	**n_{FBR}**	**Mean**	**SD**	**SEM**	**Median**	**p-values (wrt C)**	**p-values (wrt P2)**	
SD_{time} (nm)	C (Control)	18		5.12	0.55	0.13	5.23		0.002	
	P2 (Control)	18		5.89	0.68	0.16	5.59	0.002		
	P3 (Control)	18		6.02	0.78	0.18	5.93	0.001	0.65	
	C (Cyto D)	25		5.16	0.48	0.10	5.11		0.004	
	P2 (Cyto D)	25		5.66	0.68	0.14	5.58	0.004		

	P3 (Cyto D)	25		5.36	0.63	0.13	5.35	0.19	0.06	
SD_{space} (nm)	C (Control)	18		6.03	0.51	0.12	6.05		0.009	
	P2 (Control)	18		6.45	0.58	0.14	6.26	0.009		
	P3 (Control)	18		6.33	0.65	0.15	6.48	0.03	0.52	
	C (Cyto D)	25		6.51	0.62	0.12	6.48		0.53	
	P2 (Cyto D)	25		6.48	0.67	0.13	6.37	0.53		
	P3 (Cyto D)	25		6.20	0.67	0.13	6.28	0.23	0.63	
Tension (pN/µm)	C (Control)	18		68.08	21.15	4.98	64.18		0.004	
	P2 (Control)	18		49.78	17.30	4.08	48.84	0.004		
	P3 (Control)	18		44.52	15.12	3.56	38.85	3.72E-04	0.26	
	C (Cyto D)	25		59.83	14.30	2.86	56.96		0.23	
	P2 (Cyto D)	25		57.23	21.19	4.24	53.76	0.23		
	P3 (Cyto D)	25		58.65	21.12	4.22	55.99	0.35	0.80	

Normalized SD_{space} (nm)	C (Control)	18		1	0	0	1		2.18E-05	
	P2 (Control)	18		1.07	0.05	0.01	1.07	2.10E-05		
	P3 (Control)	18		1.05	0.1	0.02	1.06	1.60E-02	0.49614	
	C (Cyto D)	25		1	0	0	1		0.19847	
	P2 (Cyto D)	25		1	0.08	0.02	0.99	0.1985		
	P3 (Cyto D)	25		0.96	0.11	0.02	0.97	4.50E-03	0.15106	

**Fig S4b**										
**Parameters**	**Conditions**	**N_{cell}**	**n_{FBR}**	**Mean**	**SD**	**SEM**	**Median**	**p-values (wrt C)**	**p-values (wrt P2)**	
SD_{time} (nm)	C (Control Slow)	18	16440	5.64	1.71	0.01	5.35		<1E-5	
	P2 (Control Slow)	18	11475	6.12	1.91	0.02	5.85	<1E-5		
	P3 (Control Slow)	18	4898	6.16	1.76	0.03	5.92	<1E-5	0.008	
	C (Cyto D)	25	16126	5.29	1.38	0.01	5.13		<1E-5	
	P2 (Cyto D)	25	9693	5.82	1.29	0.01	5.69	<1E-5		
	P3 (Cyto D)	25	6375	5.47	1.2	0.02	5.32	<1E-5	0	
Relative Tension	C (Control Slow)	18	11900	2.52	5.45	0.05	1		0	
	P2 (Control	18	8308	1.63	3.9	0.0	0.71	0		

	Slow)				4	4				
	P3 (Control Slow)	18	3096	1.24	2.65	0.05	0.66	0	2.02E-05	
	C (Cyto D)	25	10991	2.75	6.57	0.06	1		<1E-5	
	P2 (Cyto D)	25	6727	1.57	3.09	0.04	0.92	<1E-5		
	P3 (Cyto D)	25	4060	1.87	4.03	0.06	0.98	0.0166	<1E-5	
SD_{space} (nm)	C (Control Slow)	18	16440	6.16	1.94	0.02	5.83		<1E-5	
	P2 (Control Slow)	18	11475	6.53	2.17	0.02	6.17	<1E-5		
	P3 (Control Slow)	18	4898	6.49	1.97	0.03	6.23	<1E-5	0.3	
	C (Cyto D)	25	16126	6.71	2.01	0.02	6.39		2.21E-05	
	P2 (Cyto D)	25	9693	6.78	1.91	0.02	6.47	2.00E-05		
	P3 (Cyto D)	25	6375	6.61	1.89	0.02	6.35	0.06	<1E-5	

**Fig S6b**										
**Parameters**	**Conditions**	**N_{cell}**	**n_{ROI}**	**Mean**	**SD**	**SEM**	**Median**	**p-values (wrt 0 min)**	**p-values (wrt 5 min)**	
Normalized Area Fraction (Transferrin with trypsin)	0 min	53	92	1.00	0.00	0.00	1.00		2.92E-15	
	5 min	53	92	2.78	4.92	0.51	1.69	2.92E-15		
	10 min	53	92	3.47	6.45	0.67	1.69	2.63E-19	0.36	
	15 min	53	92	3.61	6.00	0.63	1.77	2.92E-15		
**Parameters**	**Conditions**	**N_{cell}**	**n_{ROI}**	**Mean**	**SD**	**SEM**	**Median**	**p-values (wrt 0 min)**	**p-values (wrt 5 min)**	
Normalized Area Fraction (Transferrin without trypsin)	0 min	16	31	1.00	0.00	0.00	1.00		2.49E-03	
	5 min	16	31	1.29	0.45	0.08	1.11	2.49E-03		
	10 min	16	31	1.32	0.55	0.10	1.10	2.49E-03	0.94	
	15 min	16	31	1.34	0.66	0.12	1.16	0.49		

Fig S6c										
**Parametes**	**Conditions**	**N_{cell}**	**n_{ROI}**	**Mean**	**SD**	**SEM**	**Median**	**p-values (wrt 0 min)**	**p-values (wrt 5 min)**	
Normalized Area Fraction Rab 5	0 min	15	122	1.00	0.00	0.00	1.00		1.23E-16	
	5 min	15	122	1.42	0.92	0.08	1.26	1.23E-16		
	10 min	15	122	1.40	0.59	0.05	1.31	2.81E-20	0.75	
	15 min	15	122	1.61	0.99	0.09	1.49	2.90E-23		

FigS6d										
**Parameters**	**Conditions**	**N_{cell}**	**n_{ROI}**	**Mean**	**SD**	**SEM**	**Median**	**p-values (wrt 0 min)**	**p-values (wrt 5 min)**	**p-values (wrt 10 min)**
Normalized Area Fraction Rab 4	0 min	43	68	1.00	0.00	0.00	1.00		1.00	2.63E-19
	5 min	43	68	1.05	0.35	0.04	1.00	1.00		0.36
	10 min	43	68	1.15	0.38	0.05	1.05	0.02	0.06	
	15 min	43	68	1.13	0.47	0.06	1.02	0.21		0.76

Fig S6e										
**Parameters**	**Conditions**	**N_{cell}**	**n_{ROI}**	**Mean**	**SD**	**SEM**	**Median**	**p-values (wrt 0 min)**	**p-values (wrt 3 min)**	**p-values (wrt 10 min)**
Area Fraction clathrin pits	0 min	74	74	0.64	0.42	0.05	0.59		0.008	2.49E-03
	3 min	82	82	0.77	0.37	0.04	0.79	0.008		0.94
	6min	94	94	0.73	0.42	0.04	0.72	0.14	0.33	

Fig S7b										
**Parameters**	**Conditions**	**N_{cell}**	**n_{ROI}**	**Mean**	**SD**	**SEM**	**Median**	**p-values (wrt 0 min)**	**p-values (wrt 3 min)**	**p-valu**

										es (wrt 10 min)
Area Fraction Rab 5	0 min (control)	66	696	0.09	0.038	0.001	0.094		7.00E-04	
	3 min(Control)	58	562	0.101	0.043	0.002	0.099	7.00E-04		2.81E-20
	6 min(Control)	57	411	0.102	0.031	0.002	0.098	1.00E-04	0.62	0.75
	0 min(Dynasore)	43	386	0.13	0.053	0.003	0.119		<1E-5	
	3 min(Dynasore)	62	358	0.109	0.066	0.003	0.091	<1E-5		0.02
	6 min(Dynasore)	50	187	0.106	0.059	0.004	0.096	<1E-5	0.89	

**Parameters**	**Conditions**	**N_{cell}**	**n_{ROI}**	**Mean**	**SD**	**SEM**	**Median**	**p-values (wrt 0 min)**	**p-values (wrt 3 min)**	**p-values (wrt 10 min)**
Area Fraction Rab 4	0 min (Control)	125	125	0.339	0.025	0.002	0.337		4.00E-04	0.02
	5 min (Control)	129	129	0.352	0.03	0.003	0.352	4.00E-04		0.06
	10 min (Control)	70	70	0.301	0.058	0.007	0.304	<1E-5	<1E-5	
	0 min (Dynasore)	206	206	0.27	0.06	0.004	0.27		<1E-5	0.49
	5 min (Dynasore)	170	170	0.32	0.076	0.006	0.34	<1E-5		
	10 min (Dynasore)	103	103	0.28	0.069	0.007	0.28	0.203	3.00E-05	

Fig S7d										
**Parameters**	**Conditions**	**N_{cell}**	**n_{FBR}**	**Mean**	**SD**	**SEM**	**Median**	**p-values (wrt C)**	**p-values (wrt P2)**	
SD_{time} (nm)	C (Control)	20	3184	6.88	2.43	0.04	6.59		<1E-5	
	P2 (Control)	20	1859	8.47	2.56	0.06	8.44	<1E-5		
	P3 (Control)	20	796	7.47	2.03	0.07	7.23	<1E-5	0	
	C (Dynasore)	11	8861	6.01	1.99	0.02			<1E-5	
	P2 (Dynasore)	11	4042	7.08	2.02	0.03	6.98	<1E-5		
	P3 (Dynasore)	11	1733	5.42	1.57	0.04	5.17	0	0	
Tension (pN/µm)	C (Control)	20	2006	106.1	266.6	5.95	40.7		0	
	P2 (Control)	20	957	33.05	35.41	1.14	22.79	0		
	P3 (Control)	20	355	45.8	82.57	4.38	27.76	<1E-5	<1E-5	
	C (Dynasore)	11	6669	147.1	358.3	4.37	49.67		0	
	P2 (Dynasore)	11	3230	62.89	156.3	2.75	33.42	0		
	P3 (Dynasore)	11	1475	168.6	326.9	8.51	70.99	<1E-5	<1E-5	
SD_{space} (nm)	C (Control)	20	3184	9.17	3.63	0.06	8.84		<1E-5	
	P2 (Control)	20	1859	9.62	3.27	0.08	9.37	<1E-5		
	P3 (Control)	20	796	8.49	2.59	0.09	8.09	1.00E-04	0	
	C (Dynasore)	11	8861	7.47	2.79	0.03	7.03		0.02	
	P2 (Dynasore)	11	4042	7.29	2.57	0.04	6.98	0.02		
	P3 (Dynasore)	11	1733	6.77	2.48	0.06	6.41	0	<1E-5	

Fig S7f										
**Parameters**	**Conditions**	**N_{cell}**	**n_{FBR}**	**Mean**	**SD**	**SEM**	**Median**	**p-values (wrt C)**	**p-values (wrt P2)**	
Normalized SD_{space}	C (Control)	20	20	1	0	0	1		0.5728	
	P2 (Control)	20	20	1.04	0.11	0.02	1.03	0.5728		
	P3 (Control)	20	20	0.95	0.15	0.03	0.98	0.5728	0.081	
	C (Dynasore)	11	11	1	0	0	1		6.00E-04	

	P2 (Dynasore)	11	11	0.79	0.25	0.08	0.66	6.00E-04		
	P3 (Dynasore)	11	11	0.77	0.26	0.08	0.74	0.0076	0.8438	

Fig S7g										
**Parameters**	**Conditions**	**N_{cell}**	**n_{FBR}**	**Mean**	**SD**	**SEM**	**Median**	**p-values (wrt Control)**		
Activity	Control	11	4302	4.00E-04	9.00E-04	1.00E-05	1.00E-04			
	Dynasore	11	8861	3.00E-04	8.00E-04	9.00E-06	1.00E-04	0		

Fig S7h										
Normalized Excess Area	Control	6	3238	1.05	0.29	0.005	1			
	ML 141	17	11397	1.1	0.36	0.003	1.03	7.79E-05		

Fig S7i										
**Parameters**	**Conditions**	**N_{cell}**	**n_{FBR}**	**Mean**	**SD**	**SEM**	**Median**	**p-values (wrt C)**	**p-values (wrt P2)**	
SD_{time} (nm) Cell Wise	C (Control)	11		6.94	2.01	0.61	6.51		0.168	
	P2 (Control)	11		8.2	2.14	0.64	8.44	0.17		
	P3 (Control)	11		7.3	1.49	0.45	7.09	0.47	0.24	
	C (Dynasore)	11		5.78	1.002	0.3	5.47		0.02	
	P2 (Dyna)	11		6.89	0.83	0.25	7.09	0.02		
	P3 (Dyna)	11		5.4	0.77	0.23	5.62	0.55	0.001	
SD_{space} (nm) Cell Wise	C (Control)	11		9.54	2.75	0.83	8.78		0.84	
	P2 (Control)	11		9.48	2.501	0.75	8.06	0.84		
	P3 (Control)	11		8.43	1.77	0.54	8.13	0.39	0.26	
	C (Dynasore)	11		7.035	1.13	0.34	6.99		0.95	
	P2 (Dyna)	11		7.039	0.88	0.27	7.18	0.95		
	P3 (Dyna)	11		6.83	1.32	0.4	7.35	0.79	1	
Tension (pN/µm)	C	11		44.	22.	6.7	43.7		0.02	

Cell Wise	(Control)			32	28	2				
	P2 (Control)	11		30.93	24.55	7.4	23.14	0.02		
	P3 (Control)	11		32.78	14.75	4.45	32.5	0.26	0.36	
	C (Dynasore)	11		55.54	21.65	6.53	56.05		0.015	
	P2 (Dyna)	11		35.96	14.14	4.26	32.6	0.015		
	P3 (Dyna)	11		74.05	33.05	9.96	63.2	0.17	5.00E-04	

Fig S7j

Parameters	Conditions	N_{cell}	n_{Rois}	Mean	SD	SEM	Median	p-values (wrt Control)		
Intensity (a.u)	Control	82	18521	148.44	24.77	0.18	143.68			
	Scramble	43	7827	146.67	22.22	0.25	143.69	0.08		
	AP2	76	19094	102.43	5.52	0.04	100.69	<0.0001		

Fig S7k

Parameters	Conditions		n_{Rois}	Mean	SD	SEM	Median	p-values (wrt Control)		
Relative Abundance of protein	Control		3	1	0	0	1			
	Scramble		3	0.84	0.06	0.03	0.81	0.06		
	AP2		3	0.40	0.27	0.16	0.52	0.04		

Fig S7l

Parameters	Conditions	N_{cell}	n_{FBR}	Mean	SD	SEM	Median	p-values (wrt C)	p-values (wrt P2)	
SD_{time} (FBR wise)	C(Control)	9	7769	7.82	2.71	0.03	7.34		4.53E-17	
	P2(Control)	9	3339	8.35	2.96	0.05	7.82	4.53E-17		
	P3 (Control)	9	1130	8.29	2.06	0.06	8.25	4.25E-19	0.02	
	C (Scramble)	5	6143	5.36	1.15	0.01	5.12		7.52E-15	
	P2(Scramble)	5	1815	5.57	1.11	0.03	5.35	7.52E-15		
	P3(Scramble)	5	403	4.83	0.65	0.03	4.75	0	0	
	C (AP2)	14	7269	6.47	2.39	0.03	6.36		1.08E-52	
	P2(AP2)	14	4881	7.28	2.73	0.04	6.99	1.08E-52		

	P3 (AP2)	14	1548	8.12	2.81	0.07	7.85	6.11E-103	1.07E-27	
Relative Tension (FBR wise)	C(Control)	9	5673	2.96	7.85	0.10	1		0	
	P2(Control)	9	1942	1.91	5.96	0.14	0.65	0		
	P3 (Control)	9	685	1.90	4.13	0.16	0.88	0.98	7.31E-19	
	C (Scramble)	5	4306	2.26	4.02	0.06	1		0.001	
	P2(Scramble)	5	1166	2.21	4.15	0.12	0.89	0.001		
	P3(Scramble)	5	192	1.81	3.29	0.24	1.09	0.26	0.001	
	C (AP2)	14	5701	2.89	9.00	0.12	1		0	
	P2(AP2)	14	3454	2.02	5.49	0.09	0.81	0		
	P3 (AP2)	14	1061	1.41	4.33	0.13	0.69	0	4.17E-12	

**Fig S8b**									
**Parameters**	**Conditions**	**N_{cell}**	**n_{FBR}**	**Mean**	**SD**	**SEM**	**Median**	**p-values (wrt Control)**	**p-values (wrt P2)**
SD_{time} (nm)	C (Control)	9	8188	3.64	0.98	0.01	3.47		4.24E-73
	P2 (Control)	9	3941	4.03	1.17	0.02	3.82	4.24E-73	
	P3 (Control)	9	1145	3.59	1.03	0.03	3.44	0.04	0
	C (Dyna KO)	15	12524	3.48	1.11	0.01	3.24		3.57E-134
	P2 (Dyna KO)	15	5476	3.91	1.26	0.01	3.72	3.57E-134	
	P3 (Dyna KO)	15	2033	3.35	1.01	0.02	3.19	3.76E-5	0
**Parameters**	**Conditions**	**N_{cell}**	**n_{FBR}**	**Mean**	**SD**	**SEM**	**Median**	**p-values (wrt Control)**	**p-values (wrt P2)**
Tension (pN/µm)	C (Control)	9	5368	410.38	677.40	9.25	161.37		0
	P2 (Control)	9	2677	281.64	523.65	10.12	118.33	0	
	P3 (Control)	9	711	380.5	693.63	26.01	135.39	1.32E-11	5.62E-43
	C (Dyna KO)	15	8192	444.64	696.32	7.69	188.99		0
	P2 (Dyna KO)	15	3494	370.16	674.46	11.41	130.48	0	
	P3 (Dyna	15	133	570.	879.	24.	240.8	7.67108E-5	5.23E-5

	KO)		1	48	23	1	7		

Fig S9b									
**Parameters**	**Conditions**	**N_{cell}**	**$n_{patches}$**	**Mean**	**SD**	**SEM**	**Median**	**p-value (wrt 0 min)**	**p-value (wrt 3 min)**
Area Fraction Rab 5(ATP Dep)	0 min	26	168	0.09	0.06	0.005	0.08		0.07
	3 min	41	288	0.09	0.05	0.003	0.09	0.07	
	6min	39	255	0.08	0.05	0.003	0.09	0.52	0.17

Fig S9c									
**Parameters**	**Conditions**	**N_{cell}**		**Mean**	**SD**	**SEM**	**Median**	**p-values (wrt C)**	**p-values (wrt P2)**
SD_{time} (nm) Cell wise	C (ATP Dep.)	9		3.199	0.57	0.19	3.35		6.00E-04
	P2 (ATP Dep.)	9		6.247	1.34	0.45	6.22	6.00E-04	
	P3 (ATP Dep.)	9		3.499	0.65	0.22	3.23	0.54	6.00E-04
	C (Chol. Dep.)	16		5.598	0.84	0.211	5.39		1.00E-05
	P2(Chol.Dep.)	16		7.49	0.69	0.17	7.56	1.00E-05	
	P3(Chol.Dep.)	16		6.3	0.785	0.196	6.44	0.027	1.00E-04
	C (ATP Dep. +Chol. Dep.)	15		4.74	0.63	0.16	4.73		$<10^{-5}$
	P2 (ATP Dep. +Chol. Dep.)	15		7.628	0.9	0.23	7.5	$<10^{-5}$	
	P3 (ATP Dep. +Chol. Dep.)	15		8.014	2.14	0.55	8.44	9.66E-05	0.36
**Parameters**	**Conditions**	**N_{cell}**		**Mean**	**SD**	**SEM**	**Median**	**p-values (wrt C)**	**p-values (wrt P2)**
SD_{space} (nm) Cell wise	C (ATP Dep.)	9		9.79	0.8	0.27	9.63		0.02
	P2 (ATP Dep.)	9		8.14	1.26	0.42	8.26	0.02	
	P3 (ATP Dep.)	9		7.13	0.71	0.24	7.54	0	0.09
	C (Chol. Dep.)	16		6.72	0.67	0.17	6.66		0.78
	P2(Chol.Dep.)	16		6.83	1.19	0.3	6.98	0.78	
	P3(Chol.Dep.)	16		6.75	0.71	0.18	6.87	0.4	0.87
	C (ATP Dep. +Chol. Dep.)	15		7.83	0.44	0.11	7.8		0.17
	P2 (ATP Dep. +Chol. Dep.)	15		7.41	0.96	0.25	7.35	0.17	
	P3 (ATP Dep. +Chol. Dep.)	15		7.95	1.95	0.5	8.13	0.36	0.2
Tension (pN/μm) Cell Wise	C (ATP Dep.)	9		270.73	236.32	78.77	163.86		6.00E-04

	P2 (ATP Dep.)	9		61.27	33.21	11.07	46.28	6.00E-04	
	P3 (ATP Dep.)	9		163.87	58.797	19.599	175.56	0.479	0.001
	C (Chol. Dep.)	16		54.7	16.997	4.25	55.12		1.00E-04
	P2(Chol.Dep.)	16		31.87	6.19	1.55	32.04	1.00E-04	
	P3 Chol.Dep.)	16		43.84	13.32	3.33	40.12	0.09	0.002
	C (ATP Dep. +Chol. Dep.)	15		110.48	70.796	18.28	95.78		$<10^{-5}$
	P2 (ATP Dep. +Chol. Dep.)	15		37.497	12.24	3.16	32.6	$<10^{-5}$	
	P3 (ATP Dep. +Chol. Dep.)	15		42.13	27.16	7.01	32.94	1.00E-04	0.56

Fig S9d									
**Parameters**	**Conditions**	**N_{cell}**	**n_{FBR}**	**Mean**	**SD**	**SEM**	**Median**	**p-values (wrt C)**	**p-values (wrt P2)**
SD_{time} (nm)	C (ATP Dep.)	9	1962	3.09	0.85	0.02	2.92		0
	P2 (ATP Dep.)	9	1271	6.31	1.63	0.05	6.22	0	
	P3 (ATP Dep.)	9	550	3.33	0.81	0.03	3.19	<1E-5	0
	C (Chol. Dep.)	16	8072	5.41	1.5	0.02	5.21		0
	P2 (Chol. Dep.)	16	3438	7.62	1.83	0.03	7.47	0	
	P3 (Chol. Dep.)	16	1366	6.61	1.51	0.04	6.43	<1E-5	0
	C (ATP Dep. +Chol. Dep.)	15	8351	4.86	1.34	0.01	4.68		0
	P2 (ATP Dep. +Chol. Dep.)	15	4681	7.74	2.12	0.03	7.51	0	
	P3 (ATP Dep. +Chol. Dep.)	15	1966	7.7	2.52	0.06	7.36	0	0.05
SD_{space} (nm)	C (ATP Dep.)	9	1962	9.88	2.11	0.05	9.8		0
	P2 (ATP Dep.)	9	1271	8.26	2.07	0.06	8.07	0	
	P3 (ATP Dep.)	9	550	7.46	1.65	0.07	7.42	0	<1E-5
	C (Chol. Dep.)	16	8072	6.89	2.34	0.03	6.44		0.15
	P2 (Chol. Dep.)	16	3438	6.99	2.51	0.04	6.57	0.15	
	P3 (Chol. Dep.)	16	13	7.07	2.1	0.0	6.71	3.84E-05	0.01

			66		9	6			
	C (ATP Dep. +Chol. Dep.)	15	8351	8.25	2.8	0.03	7.81		<1E-5
	P2 (ATP Dep. +Chol. Dep.)	15	4681	8.02	2.83	0.04	7.54	<1E-5	
	P3 (ATP Dep. +Chol. Dep.)	15	1966	8.04	2.72	0.06	7.59	0.004	0.45
**Parameters**	**Conditions**	**N_{cell}**	**n_{FBR}**	**Mean**	**SD**	**SEM**	**Median**	**p-values (wrt C)**	**p-values (wrt P2)**
Relative Tension	C (ATP Dep.)	9	1363	2.77	4.65	0.13	1		0
	P2 (ATP Dep.)	9	1014	0.37	0.48	0.02	0.24	0	
	P3 (ATP Dep.)	9	390	1.31	1.97	0.1	0.75	<1E-5	<1E-5
	C (Chol. Dep.)	16	6032	2.32	6.01	0.08	1		0
	P2 (Chol. Dep.)	16	2291	0.71	0.83	0.02	0.53	0	
	P3 (Chol. Dep.)	16	1087	1	1.69	0.05	0.69	0	<1E-5
	C (ATP Dep. +Chol. Dep.)	15	7169	2.68	5.07	0.06	1		0
	P2 (ATP Dep. +Chol. Dep.)	15	4281	0.59	0.91	0.01	0.4	0	
	P3 (ATP Dep. +Chol. Dep.)	15	1677	0.97	2.48	0.06	0.41	0	0.002

Fig S10e									
**Parameters**	**Conditions**	**N_{cell}**	**n_{ROI}**	**Mean**	**SD**	**SEM**	**Median**	**p-values (wrt 0 min)**	
No of tubules (µm-1) (Chol dep)	0 min	10	176	0.03	0.01	7.70E-04	0.03		
	3 min	9	113	0.01	0.01	0.0009	0.01	0.85	

Fig S10f									
**Parameters**	**Conditions**	**N_{cell}**	**n_{ROI}**	**Mean**	**SD**	**SEM**	**Median**	**p-values (wrt 0 min)**	
No of tubules (µm-1) (Control)	0 min	8	97	0.01	0.01	0.00	0.01		
	3 min	11	152	0.02	0.01	9.75E-04	0.02	<0.0001	

Fig S10g									
**Parameters**	**Conditions**	**N_{cell}**	**n_{ROI}**	**Mean**	**SD**	**SEM**	**Median**	**p-values (wrt Control)**	
No of tubules (µm-1)	Control (0 min)	10	176	0.03	0.01	0.00	0.03		
	ATP Dep (0 min)	20	13	0.45	0.2	0.02	0.43	<0.0001	

			9		5				
	Chol dep (0 min)	8	97	0.01	0.01	0.00	0.01	<0.0001	
	ATP Dep + Chol Dep (0 min)	21	149	0.49	0.21	0.02	0.48	<0.0001	
	Control (3 min)	11	152	0.02	0.01	0.0009	0.02		
	ATP Dep (3 min)	18	124	0.58	0.25	0.02	0.59	<0.0001	
	Chol dep (3 min)	9	113	0.01	0.01	0.0009	0.01	<0.0001	
	ATP Dep + Chol Dep (3 min)	24	161	0.41	0.27	0.02	0.39	<0.0001	

**Table S2**				
Values obtained from LMM analysis of FBR-wise comparisons				
**Normal Slow - tension**				
	**p-value**	**slope**	**Standard error**	
C-P2	0.007	-0.26	0.1	
C-P3	$<10^{-5}$	-0.55	0.1	
P2-P3	0.001	-0.29	0.09	
**Normal Slow - SD_{time}**				
C-P2	0.009	0.07	0.03	
C-P3	$<10^{-5}$	0.16	0.03	
P2-P3	0.001	0.09	0.03	
**Normal Slow - SD_{space}**				
C-P2	0.262	0.02	0.02	
C-P3	0.132	0.04	0.03	
P2-P3	0.509	0.02	0.03	
	**p-value**	**slope**	**Standard error**	
**Normal Fast - Tension**				
C-P2	0.001	-0.44	0.14	
C-P3	0.0003	-0.35	0.1	
P2-P3	0.492	0.1	0.14	
**Normal Fast - SD_{time}**				
C-P2	0.0001	0.15	0.04	
C-P3	0.0009	0.13	0.04	

P2-P3	0.672	-0.02	0.04	
**Normal Fast - SD_{space}**				
C-P2	0.921	0	0.04	
C-P3	0.785	-0.01	0.02	
P2-P3	0.562	-0.02	0.03	
**Dynasore – tension**				
C-P2	0.002	-0.38	0.13	
C-P3	0.019	0.32	0.14	
P2-P3	$<10^{-5}$	0.7	0.11	
**Dynasore - SD_{time}**				
C-P2	0.0005	0.14	0.04	
C-P3	0.109	-0.07	0.05	
P2-P3	2.28E-08	-0.21	0.04	
**Dynasore - SD_{space}**				
C-P2	0.648	0.01	0.03	
C-P3	0.324	-0.04	0.04	
P2-P3	0.112	-0.06	0.03	
**Cyto D - SD_{time}**				
C-P2	0.007	0.06	0.02	
C-P3	0.642	0.01	0.02	
P2-P3	0.014	-0.05	0.02	
**Cyto D - SD_{space}**				
C-P2	0.13	-0.03	0.02	
C-P3	0.005	-0.07	0.03	
P2-P3	0.031	-0.04	0.02	
	**p-value**	**slope**	**Standard error**	
**Cyto D - tension**				
C-P2	0.576	-0.04	0.07	
C-P3	0.965	0	0.06	
P2-P3	0.562	0.04	0.07	
**ATP depletion - SD_{time}**				
C-P2	3.74E-10	0.54	0.09	
C-P3	0.0005	0.19	0.05	
P2-P3	0.0001	-0.35	0.09	

**ATP depletion - SD$_{space}$**				
C-P2	1.46E-12	-0.17	0.02	
C-P3	8.16E-20	-0.35	0.04	
P2-P3	0.0018	-0.18	0.06	
**ATP depletion - tension**				
C-P2	1.49E-31	-1.55	0.13	
C-P3	0.0003	-0.62	0.17	
P2-P3	1.81E-07	0.93	0.18	
**ATP+ cholesterol depletion - SD$_{time}$**				
C-P2	1.61E-40	0.47	0.04	
C-P3	1.16E-33	0.54	0.04	
P2-P3	0.123	0.07	0.05	
**ATP+ cholesterol depletion - SD$_{space}$**				
C-P2	3.75E-08	-0.73	0.13	
C-P3	3.45E-09	-0.9	0.15	
P2-P3	0.0002	-0.17	0.05	
**ATP+ cholesterol depletion – tension**				
C-P2	7.58E-44	-1.16	0.08	
C-P3	1.74E-62	-1.3	0.08	
P2-P3	0.024	-0.15	0.07	
**Cholesterol Depletion - tension**				
C-P2	1.17E-18	-0.57	0.06	
C-P3	0.0003	-0.25	0.07	
P2-P3	1.07E-10	0.31	0.05	
	**p-value**	**slope**	**Standard error**	
**Cholesterol Depletion - SD$_{time}$**				
C-P2	1.30E-22	0.28	0.03	
C-P3	0.0003	0.12	0.03	
P2-P3	6.79E-10	-0.16	0.03	
**Cholesterol Depletion - SD$_{space}$**				
C-P2	0.279	0.03	0.03	
C-P3	0.742	-0.01	0.02	
P2-P3	0.235	-0.03	0.03	
**Dynamin Mutant SD$_{time}$**				
C-P2	2.25E-05	0.13	0.03	
C-P3	0.46	-0.03	0.04	

P2-P3	2.82E-05	-0.16	0.04	
**Dynamin Mutant Tension**	**p-value**	**slope**	**Standard error**	
C-P2	5.19E-03	-0.29	0.10	
C-P3	0.03	0.24	0.11	
P2-P3	4.17E-05	0.53	0.13	
**Excess Area**	**p-value**	**slope**	**Standard error**	
**C(Ctrl)-C(Dynasore)**				
7 sets of ctrl, 3 sets of dynasore	6.43E-11	0.22	0.03	
**AP2 sirna Tension**				
C-P2	0.042	-0.1	0.057	
C-P3	0.0009	-0.31	0.09	
P2-P3	0.003	-0.21	0.07	
**AP2 sirna Sdtime**				
C-P2	0.09	0.05	0.03	
C-P3	0.01	0.13	0.05	
**TrypLE Tension**				
C-P2	6.60E-17	-0.47	0.06	
C-P3	0.0004	-0.24	0.07	
P2-P3	0.00	0.24	0.06	
**EDTA Tension**				
C-P2	1.45E-18	-0.32	0.04	
C-P3	7.79E-07	-0.20	0.04	
P2-P3	0.001	0.12	0.04	
**Scramble Tension**				
C-P2	0.13	-0.12	0.08	
C-P3	0.58	0.04	0.08	
P2-P3	0.08	0.14	0.08	
**Control of AP2 siRNA Tension**				
C-P2	0.01	-0.13	0.05	
C-P3	0.59	-0.04	0.07	
P2-P3	0.21	0.12	0.10	
**Control of ATP Dep SD_{time}**				
C-P2	0.01	0.11	0.04	
C-P3	0.58	0.04	0.06	
P2-P3	0.04	-0.08	0.04	

Extended Methods:

Details about Linear Mixed Model:

We implement a Linear Mixed Model (LMM) using the following formula (Eq. 1):

Value ~ Phase +(Phase|Set:Cell)' (Eq 1)

The measured parameter (Value, representing SD_{time} or SD_{space} log (excess area) or log(tension)) was considered as a function of de-adhesion phase. Predictor variables - set number of experiment (Set) and cell number (Cell) - are grouping variables. Cell nested in Set contribute random intercepts (with possible correlation with random slope) to the model to control for the variation across sets, treat cells to be grouped under sets and FBRs to be grouped under cells and thus account of the repeated measurements in clusters. The Linear Mixed Model class from the Statistics Toolbox in MATLAB was used for model fitting. The linear mixed model coefficients were estimated using maximal likelihood (ML) as the default settings.
